# Strigolactones enhance apple drought resistance via the MsABI5-MsSMXL1-MsNAC022 cascade

**DOI:** 10.1093/hr/uhaf101

**Published:** 2025-04-09

**Authors:** Xiang Zhang, BingYang Du, Maihemuti Turupu, Yuqin Xiao, Qisheng Yao, Shilin Gai, Qiaoqiao Zhang, Xinyu Wang, Yongzhen Yan, Zhengyang Wen, Shuo Wang, Wenjun Lu, Pengtao Yue, Tianhong Li

**Affiliations:** Frontiers Science Center for Molecular Design Breeding, College of Horticulture, China Agricultural University, No.2 Yuanmingyuan West Road, Haidian District, Beijing 100193, China; Frontiers Science Center for Molecular Design Breeding, College of Horticulture, China Agricultural University, No.2 Yuanmingyuan West Road, Haidian District, Beijing 100193, China; Frontiers Science Center for Molecular Design Breeding, College of Horticulture, China Agricultural University, No.2 Yuanmingyuan West Road, Haidian District, Beijing 100193, China; Frontiers Science Center for Molecular Design Breeding, College of Horticulture, China Agricultural University, No.2 Yuanmingyuan West Road, Haidian District, Beijing 100193, China; Frontiers Science Center for Molecular Design Breeding, College of Horticulture, China Agricultural University, No.2 Yuanmingyuan West Road, Haidian District, Beijing 100193, China; Frontiers Science Center for Molecular Design Breeding, College of Horticulture, China Agricultural University, No.2 Yuanmingyuan West Road, Haidian District, Beijing 100193, China; Frontiers Science Center for Molecular Design Breeding, College of Horticulture, China Agricultural University, No.2 Yuanmingyuan West Road, Haidian District, Beijing 100193, China; Frontiers Science Center for Molecular Design Breeding, College of Horticulture, China Agricultural University, No.2 Yuanmingyuan West Road, Haidian District, Beijing 100193, China; Frontiers Science Center for Molecular Design Breeding, College of Horticulture, China Agricultural University, No.2 Yuanmingyuan West Road, Haidian District, Beijing 100193, China; Frontiers Science Center for Molecular Design Breeding, College of Horticulture, China Agricultural University, No.2 Yuanmingyuan West Road, Haidian District, Beijing 100193, China; Frontiers Science Center for Molecular Design Breeding, College of Horticulture, China Agricultural University, No.2 Yuanmingyuan West Road, Haidian District, Beijing 100193, China; Frontiers Science Center for Molecular Design Breeding, College of Horticulture, China Agricultural University, No.2 Yuanmingyuan West Road, Haidian District, Beijing 100193, China; Frontiers Science Center for Molecular Design Breeding, College of Horticulture, China Agricultural University, No.2 Yuanmingyuan West Road, Haidian District, Beijing 100193, China; Frontiers Science Center for Molecular Design Breeding, College of Horticulture, China Agricultural University, No.2 Yuanmingyuan West Road, Haidian District, Beijing 100193, China

## Abstract

Drought stress limits plant growth, development, and yield in apple (*Malus*). Strigolactones (SLs) work with abscisic acid (ABA) to improve drought resistance in plants, but how this synergistic mechanism functions remains unclear. Here, we determined that SLs promote drought resistance in apple in an *ABSCISIC ACID INSENSITIVE5* (*MsABI5*)-related manner. During drought stress of a wild apple species (*Malus sieversii*), SLs enhanced the expression of *MsABI5*, encoding a major transcription factor involved in ABA signaling. MsABI5 bound to the promoter of the gene encoding delta-1-pyrroline-5-carboxylate synthase (*MsP5CS2.2*), upregulating its expression and thereby enhancing proline accumulation and drought resistance. In addition, MsABI5 suppressed the expression of *MsSMXL1*, encoding a major transcriptional repressor involved in SL signaling. MsSMXL1 interacted with MsNAC022 instead of MsABI5 to repress the transactivation activity of MsNAC022. *MsNAC022* was upregulated by MsABI5, and MsNAC022 directly promoted *MsP5CS2.2* expression to enhance proline accumulation and drought resistance. These findings suggest that MsSMXL1 and MsNAC022 comprise a regulatory node downstream of MsABI5 during drought stress in apple. Together, our findings suggest that in apple, SLs increase drought resistance by activating the MsABI5-MsSMXL1-MsNAC022 cascade.

## Introduction

Drought stress limits plant growth and development leading to chlorophyll degradation and a burst of reactive oxygen species (ROS) accumulation. These responses reduce photosynthesis, induce membrane peroxidation, and suppress plant growth; long-term severe drought stress can cause plant death [1, 2]. Plants have developed a variety of mechanisms to protect against drought stress. Under drought conditions, plants rapidly accumulate osmotic substances, such as proline, to increase cellular water potential, thereby sustaining *in vivo* water levels [3, 4]. Proline is a multifunctional amino acid that acts as a protecting compound and metabolic signal during drought stress; it is involved in improvement of photosynthesis by maintaining the ratio of oxidized NADP/NADPH in the shoot and leaf, and promotion of redox balances by scavenging ROS and reducing oxidative damage, thereby improving drought tolerance [5, 6].

Apple (*Malus domestica*) is an economically important horticultural crop cultivated in temperate areas worldwide that often suffers from drought stress [7]. *Malus sieversii*, the ancestral apple species of modern apple cultivars, has become the excellent drought-resistant rootstock resources for apple and initial gene pool for genetic improvement of apple drought-resistant rootstock due to its highly developed root and vascular system, and extremely strong drought resistance [8, 9]. In apple, drought stress also activates stress responses associated with the phytohormones that in turn enhance drought resistance [10, 11].

Strigolactones (SLs) are a new class of phytohormones derived from carotenoids and regulates lateral shoot branching or root growth [12, 13]. SL signaling compounds also enhance drought resistance [14–20]. For instance, treatment with the synthetic SLs *rac*-GR24 improve drought tolerance by increasing the relative leaf water content in wheat [21] and by alleviating chlorophyll degradation in plants of grape (*Vitis vinifera*) [22]. SLs might improve drought resistance by cooperating with ABA signaling and altering plant sensitivity to ABA [23, 24]. In tomato (*Solanum lycopersicum*), SL-depleted plants show increased sensitivity to drought stress due to the hyposensitivity of their stomata to ABA [25]. In SL-deficient Arabidopsis mutants, ABA-mediated stomatal closure in leaves is inhibited, increasing water loss and reducing drought tolerance. ABA treatment mitigates this phenotype, suggesting that SL-deficient mutants have reduced sensitivity to ABA [26]. SUPPRESSOR OF MAX2-LIKE (SMXL)/DWARF53 (D53) is the central transcriptional suppressor of SL signal transduction [13]. In the absence of SLs, SMXLs interact with TFs to sequester their transcriptional activity, thereby suppressing SL-associated effects. When SLs are present, MORE AXILLARY GROWTH 2 (MAX2; an F-box protein involved in SL signaling) and D14, which are involved in forming the SLs-D14-SCF^MAX2^ ubiquitin complex, interact with SMXLs to trigger their ubiquitin-mediated degradation by 26S proteasome, thereby leading to release the TFs suppressed by SMXL proteins with EAR motif, an effect induced by SLs [13, 27–31]. SMXLs play important roles in activating SL signaling. In Arabidopsis, the degradation of SMXL proteins depended on MAX2 and D14 is activated by *rac*-GR24 [29, 30, 32] and releases the *BRC1*, *TCP1*, and *PAP1*, thereby repressing shoot branching, stimulating leaf elongation, and promoting anthocyanin accumulation through the activated SL signaling cascades [13].

ABA regulates plant growth and seed germination and is crucial for many stress resistance processes, especially resistance to drought stress [33–35]. Many transcription factors (TFs) that respond to ABA regulate drought tolerance. For example, in wheat (*Triticum aestivum*), the NAC (NAM ATAF1/2 and CUC2) TF TaSNAC8-6A is activated by ABA and positively regulates drought resistance [36]. In various monocot and dicot species, ABI5 and ABA-responsive element binding factors (ABFs), the key TFs in ABA signaling, also regulate drought resistance [37–41]. In trifoliate orange (*Poncirus trifoliata*), PtrABF2 enhances drought tolerance by promoting the accumulation of polyamines, which help plants adapt to drought stress [42]. In cotton, GhABF3 induces the expression of *GhRD29B* to improve drought resistance [43]. Moreover, ABI5 is not only the ABA response factor but can act as the multifunctional regulators in improving drought resistance through multiple molecular and metabolic pathways. In various species, ABI5 directly promotes the expression of drought-responsive genes, including *RESPONSE TO DESICCATION 29A* (*RD29A*), *RD22*, and *DEHYDRATION RESPONSIVE ELEMENT BINDING 1A* (*DREB1A*), to improve ABA-mediated drought tolerance [44–46]. Arabidopsis (*Arabidopsis thaliana*), cotton (*Gossypium hirsutum*), and rice (*Oryza sativa*) plants overexpressing *ABI5* display enhanced resistance to drought owing to their lower chlorophyll degradation and hydrogen peroxide accumulation when subjected to drought stress [37, 47–49]. In apple, the transcriptional activity of MdABI5 is repressed by TEOSINTE BRANCHED 1/CYCLOIDEA/PCF 46 (TCP46), which results in minimal *RD29A* expression and poor drought tolerance [50]. During drought stress, however, apple plants overexpressing *MdABI5* accumulate less malondialdehyde (MDA) but more proline than wild-type seedlings due to upregulation of the proline biosynthetic gene encoding *delta-1-pyrroline-5-carboxylate synthase* (*P5CS*), thus enhancing drought resistance [46, 51].

Several studies have explored the interaction between SLs and ABA during drought stress and the basic functions of ABI5 in ABA-mediated drought resistance [25, 50]. However, it remains unclear whether SMXLs associated with SL signaling contribute to drought resistance by interacting with an ABI5-mediated pathway in apple. In this study, we showed that treatment with the synthetic SLs *rac*-GR24 improve drought tolerance in apple plants in an MsABI5-related manner. MsABI5 directly activates the proline biosynthetic gene *MsP5CS2.2* to enhance proline accumulation and drought tolerance. The drought-responsive MsSMXL1 does not directly interact with MsABI5, but expression of *MsSMXL1* is negatively regulated by MsABI5, and the overexpression of *MsSMXL1* leads to greater sensitivity to drought stress. By contrast, MsSMXL1 directly interacts with MsNAC022, a TF upregulated by MsABI5, to suppress its transactivation activity; MsNAC022 directly upregulates *MsP5CS2.2* expression. Our findings reveal a pathway by which SLs improve drought tolerance by enhancing the responses downstream of MsABI5.

## Results

### SLs enhance drought tolerance in apple

**Figure 1:**
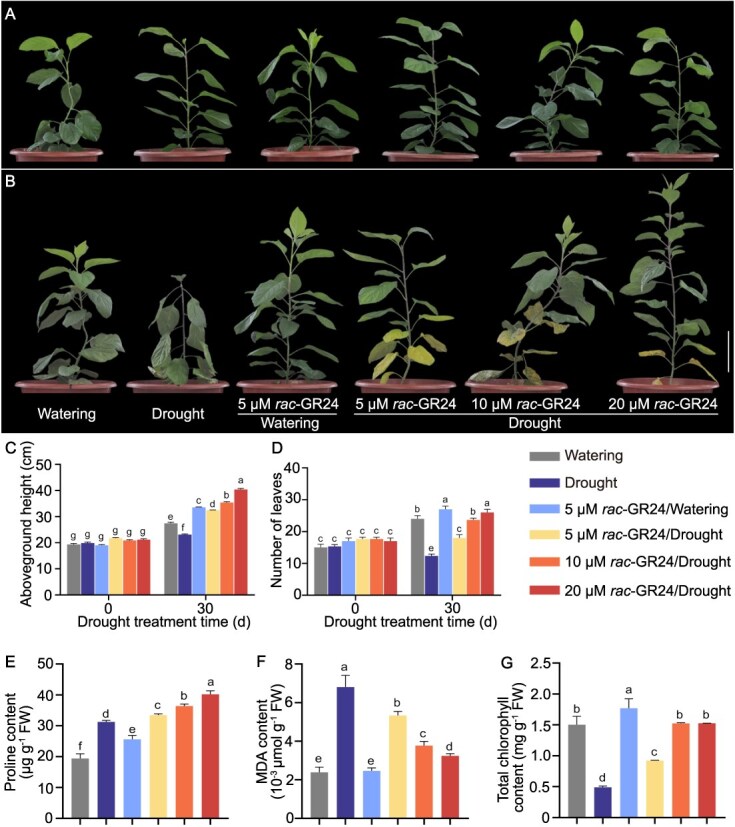
SLs treatment enhance drought resistance in apple. A and B Wild apple (*Malus sieversii*) plants at similar stages of growth (14–16 leaves, 18–21 cm height) were selected for treatment (A, phenotype before treatment). The plants were treated with *rac*-GR24 (5, 10, 20 μM) and grown without watering until soil volumetric water content reached 0% (*rac*-GR24/Drought). Plants subjected to normal watering with soil volumetric water content maintained at 40%–45% (Watering) or without watering until soil volumetric water content reached 0% (Drought) and plants treated with 5 μM *rac*-GR24 and normal watering with soil volumetric water content maintained at 40%–45% (*rac*-GR24/Watering) were used as controls (B, phenotypes of drought treatment). Scale bar, 10 cm. C and D Aboveground height and leaf number before and after treatment. The *x*-axis indicates days (d) after treatment. Three apple plants were used per treatment, an independent measurement of aboveground height and leaf number of *M. sieversii* plant from each treatment was used as one biological replicate. Three replicates were performed in this determination. Proline (E), MDA (F), and total chlorophyll contents (G) were measured after treatment. Three leaves each from the top, middle, and bottom of each plant were collected separately; a total of 27 leaves from three plants were collected for each treatment and evenly divided into three sets as three biological replicates per treatment. One independent proline, MDA, or chlorophyll measurement from each biological replicate was considered one technical replicate; three replicates were performed in this measurement. MDA, malondialdehyde. FW, fresh weight. Values represent means ± SD. Different letters (a–g) indicate significance differences as determined by LSD range test (*P* < 0.05).

To explore the effect of SLs on drought tolerance in apple, we treated wild apple (*M. sieversii*) plants with *rac*-GR24 (5, 10, and 20 μM) and grew them without watering to induce drought stress (*rac*-GR24/Drought). Plants with normal watering (Watering) or without watering (Drought) and plants treated with *rac*-GR24 and normal watering (5 μM *rac*-GR24/Watering) were used as controls (Fig. 1A and B). Drought stress suppressed plant growth and decreased leaf number, and *rac*-GR24 treatment relieved the inhibited plant growth caused by drought stress (Fig. 1C and D). To investigate the responses of apple plants to drought stress, we quantified proline, MDA, and chlorophyll levels. Both drought and *rac*-GR24/Watering treatment increased proline levels compared with watering treatment, and these were further enhanced in *rac*-GR24/Drought-treated plants compared with drought-treated plants (Fig. 1E). Drought-treated plants accumulated significantly higher levels of MDA than *rac*-GR24/Drought-treated plants (Fig. 1F). Drought stress led to a severe decrease in chlorophyll levels compared with normal watering treatment, and *rac*-GR24 treatment relieved this drought-induced chlorophyll loss (Fig. 1G). These results indicate that SLs can enhance drought tolerance in apple by promoting proline accumulation and reducing MDA accumulation and chlorophyll degradation.

### SLs activate *MsABI5* to enhance drought tolerance in apple

**Figure 2:**
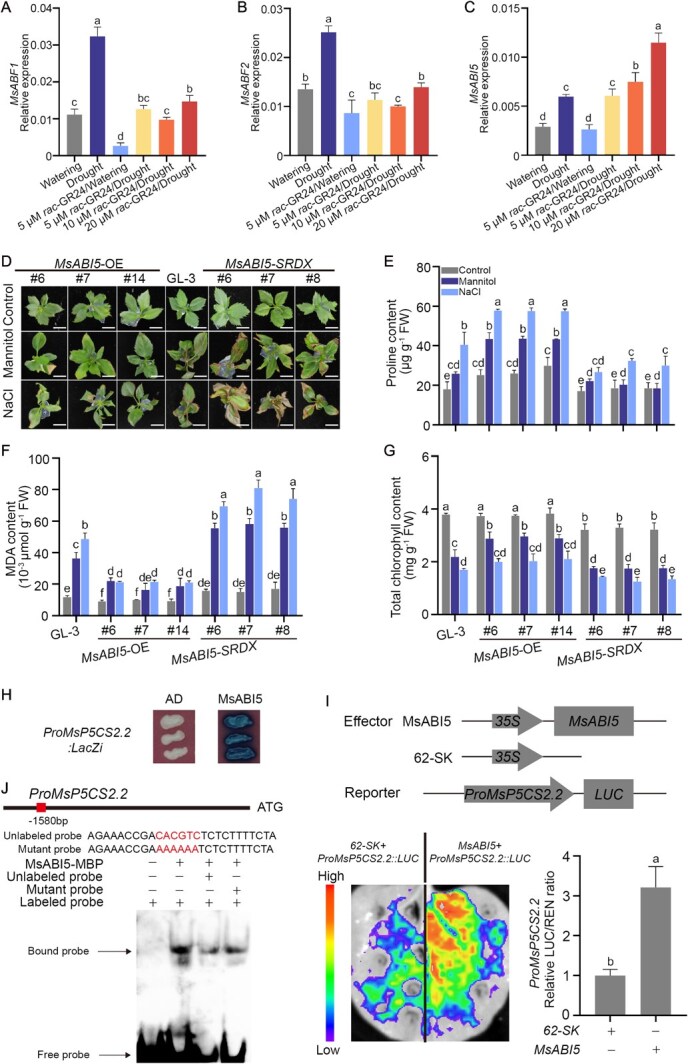
MsABI5 improves proline accumulation and osmotic stress resistance in apple. Effects of *rac-*GR24 on the expression of *MsABF1* (A), *MsABF2* (B) and *MsABI5* (C) during drought stress. D Apple (*Malus domestica*) plants overexpressing *MsABI5* (*MsABI5*-OE) or expressing a repressor version of *MsABI5* (*MsABI5-SRDX*) and ‘GL-3’ plants were treated with mannitol or NaCl to induce osmotic stress for 14 days. Untreated plants were used as controls. Scale bars, 1 cm. E–G Proline, MDA, and chlorophyll contents were measured in *MsABI5*-OE, *MsABI5-SRDX*, or ‘GL-3’ plants. Nine apple plants were used per treatment, three apple plants were used as one biological replicate, and three biological replicates were performed for each treatment. Three independent measurements were performed for proline, MDA, and chlorophyll contents. The *x*-axis indicates the transgenic line numbers. FW, fresh weight. Values represent means ± SD. Different letters (a–f) indicate significant differences as determined by LSD range test (*P* < 0.05). H MsABI5 binds to the *MsP5CS2.2* promoter. Y1H assays were performed by co-transforming yeast with a plasmid expressing *MsABI5* and a plasmid driven by the *MsP5CS2.2* promoter. The empty pB42AD vector (AD) and a plasmid containing the *MsP5CS2.2* promoter were used as controls. I MsABI5 positively regulates *MsP5CS2.2* promoter activity. MsABI5 effector and *MsP5CS2.2* promoter-LUC reporter plasmids were co-expressed in *N. benthamiana* leaves to analyze LUC activity. Three independent replicates were performed. Values represent means ± SD. Different letters (a and b) indicate significant differences by LSD range test (*P* < 0.05). J MsABI5 binds to the *MsP5CS2.2* promoter. Electrophoretic mobility shift assays (EMSAs) were conducted with a biotin-labeled *MsP5CS2.2* promoter fragment containing an ABA-response element (ABRE). An unlabeled version of the same *MsP5CS2.2* promoter fragment was used as an unlabeled competitor. For the mutant probe, the ABRE was mutated into AAAAAA and used as an unlabeled competitor.

SLs can promote drought resistance by increasing the sensitivity of plants to ABA [25, 52]. To investigate how SLs influence ABA responses, we searched the apple genome for genes encoding TFs that serve as master regulators of ABA signaling according to the GDR (https://www.rosaceae.org/) and TAIR (https://www.arabidopsis.org/) databases. We identified *ABI5* and two *ABF* genes and quantified their expression in *rac*-GR24-treated *M. sieversii* plants during drought stress using reverse-transcription quantitative PCR (RT-qPCR) to determine whether any of these genes participate in SLs-mediated drought resistance. *MsABF1* and *MsABF2* were upregulated by drought stress but downregulated by *rac*-GR24/Watering treatment compared with the normal watering treatment; there was little difference in expression between the watering treatment and the *rac*-GR24/Drought treatment (Fig. 2A and B). Drought stress significantly promoted *MsABI5* expression, but expression of this gene did not significantly differ between plants that were watered normally and plants treated with *rac*-GR24 plus watering (Fig. 2C).

During drought stress, *rac*-GR24 treatment enhanced the transcription of *MsABI5* (Fig. 2C), suggesting that *MsABI5* might play a role in SLs-enhanced drought resistance. We expressed an *MsABI5-GFP* fusion gene driven by the *35S* promoter in ‘GL-3’ (*M. domestica*) using *Agrobacterium*-mediated transformation to generate *MsABI5* overexpression lines (*MsABI5*-OE). We also ligated the coding sequence of the SRDX transcriptional repressor domain [53], downstream of the coding sequence of *MsABI5* and expressed this *MsABI5-SRDX* fusion gene driven by the *35S* promoter in ‘GL-3’ to create lines harboring a version of MsABI5 that should repress transcription at its target sites (MsABI5-SRDX) (Fig. S1, see online supplementary material).

To investigate whether MsABI5 regulates drought resistance in apple, we treated *MsABI5*-OE and *MsABI5-SRDX* apple plants with mannitol or NaCl to induce osmotic stress and compared their sensitivity to drought; untreated plants were used as controls. The growth of *MsABI5*-OE, *MsABI5-SRDX*, and control ‘GL-3’ apple plants was similar in the absence of osmotic stress (Fig. 2D). However, *MsABI5*-OE apple plants showed more tolerance of osmotic stress (imposed by mannitol or NaCl treatment) than ‘GL-3’ plants (Fig. 2D). By contrast, osmotic stress resistance was attenuated in *MsABI5-SRDX* apple plants compared with those of ‘GL-3’ (Fig. 2D). We measured proline, MDA, and chlorophyll accumulation in these lines. In the absence of osmotic stress, proline accumulated to higher levels in *MsABI5*-OE apple plants than in ‘GL-3’ plants, but a significant difference was not observed between *MsABI5-SRDX* and ‘GL-3’ apple plants (Fig. 2E). MDA and chlorophyll levels were similar between *MsABI5*-OE and ‘GL-3’ apple plants, but higher MDA and lower chlorophyll levels were observed in *MsABI5-SRDX* apple plants than in ‘GL-3’ plants (Fig. 2F and G). During osmotic stress, proline accumulated at much higher levels in *MsABI5*-OE apple plants than in ‘GL-3’ plants, whereas proline accumulation was suppressed in *MsABI5-SRDX* apple plants (Fig. 2E). MDA accumulation and chlorophyll degradation were lower in the *MsABI5*-OE lines but higher in the *MsABI5-SRDX* lines compared with those in ‘GL-3’ (Fig. 2F and G). These results indicate that SLs-activated MsABI5 enhances osmotic stress resistance in apple by promoting proline accumulation and inhibiting MDA accumulation and chlorophyll degradation.

Proline is important for maintaining internal water levels in plants during drought stress. DELTA 1-PYRROLINE-5-CARBOXYLATE SYNTHASE (P5CS) catalyzes the key step in proline biosynthesis [51, 54]. Drought stress generally causes the occurrence of osmotic stress [4]. Treatment with 1.2 M mannitol successfully induced obvious drought stress in *M. sieversii* plants (Fig. S2A, see online supplementary material), and endogenous ABA levels increased significantly under this treatment (Fig. S2B, see online supplementary material). We identified three *P5CS* genes, *P5CS2.1/2.2/2.3*, in the apple genome using the GDR and TAIR databases and measured their transcript levels in *M. sieversii* plants treated with mannitol to induce osmotic stress. All three genes were induced by osmotic stress, and this coincided with *MsABI5* expression and proline accumulation (Fig. S2C–G, see online supplementary material). To explore whether MsABI5 regulates the expression of *P5CS* genes, we compared their expression among *MsABI5*-OE lines, *MsABI5-SRDX* lines, and ‘GL-3’. *MdP5CS2.1/2.2/2.3* were upregulated in *MsABI5*-OE lines compared with ‘GL-3’, whereas significant differences in expression were not observed between *MsABI5-SRDX* and ‘GL-3’ plants in the absence of osmotic stress (Fig. S3, see online supplementary material). When these apple plants were exposed to osmotic stress, *MdP5CS2.1/2.2/2.3* were significantly upregulated in *MsABI5*-OE apple plants but repressed in *MsABI5-SRDX* apple plants compared with ‘GL-3’ plants (Fig. S3, see online supplementary material).

The expression patterns of *P5CS2.1/2.2/2.3* and *MsABI5* were similar, and *P5CS2.2* was the most highly expressed among the three *P5CS* genes; we, therefore, chose *P5CS2.2* for further study. We investigated whether MsABI5 binds to the *MsP5CS2.2* promoter using a yeast one-hybrid (Y1H assay), which confirmed the interaction between MsABI5 and the *MsP5CS2.2* promoter (Fig. 2H). We explored the effect of MsABI5 in regulating *MsP5CS2.2* promoter activity using dual-luciferase (dual-LUC) activity assays. When *MsABI5* and *ProMsP5CS2.2:LUC* were co-expressed in *Nicotiana benthamiana* leaves, we observed significantly higher chemiluminescence and a higher LUC ratio in the presence of MsABI5 (Fig. 2I), indicating that MsABI5 positively regulates *MsP5CS2.2* promoter activity. To further test this binding, we performed electrophoretic mobility shift assays (EMSAs). MsABI5 bound to the ABA-response element (ABRE) in the *MsP5CS2.2* promoter, and the mobility shift was impaired by unlabeled competitor (Fig. 2J). These results indicate that MsABI5 promotes proline accumulation during drought stress by directly upregulating *MsP5CS2.2* expression.

**Figure 3:**
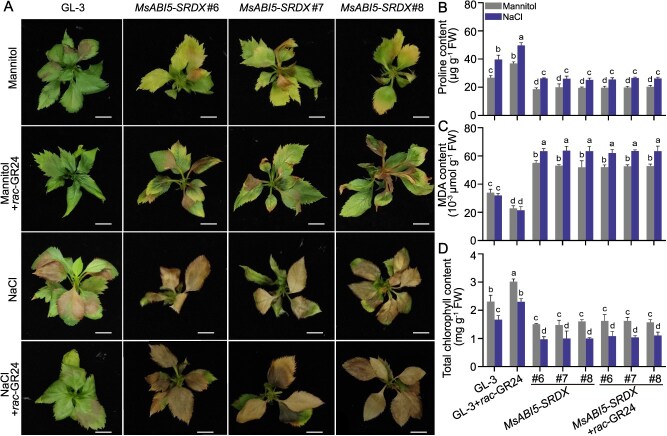
SLs enhance osmotic stress resistance in apple in an MsABI5-related manner. A Apple plants expressing a repressor version of *MsABI5* (*MsABI5-SRDX*) and ‘GL-3’ plants were treated with *rac*-GR24 and subjected to mannitol or NaCl treatment to induce osmotic stress for 14 days. The drought sensitivity among these apple plants was then compared. Apple plants not treated with *rac*-GR24 were used as controls. Scale bars, 1 cm. B–D Proline, MDA, and chlorophyll contents were measured in *MsABI5-SRDX* or ‘GL-3’ plants. Nine apple plants were used per treatment, three apple plants were used as one biological replicate, three biological replicates were performed for each treatment, and one representative apple plant from each treatment is pictured. Three independent measurements were performed for proline, MDA, and chlorophyll contents from three biological replicates. The *x*-axis indicates the transgenic line numbers. FW, fresh weight. Values represent means ± SD. Different letters (a-d) indicate significant differences as determined by LSD range test (*P* < 0.05).

To explore whether MsABI5 plays a central role in SLs-mediated drought tolerance in apple, we treated *MsABI5-SRDX* apple plants with *rac*-GR24 and used mannitol or NaCl to induce osmotic stress. Mannitol or NaCl treatment induced leaf yellowing or senescence in ‘GL-3’ plants, and *rac*-GR24 treatment alleviated the damage to leaves caused by osmotic stress (Fig. 3A). When function of MsABI5 was inhibited, *rac*-GR24 treatment failed to rescue the osmotic stress-induced damage to leaves, failed to promote proline accumulation, and inhibit MDA accumulation or chlorophyll degradation during osmotic stress (Fig. 3B–D). These results indicate that SLs-enhanced drought resistance in apple is related to MsABI5.

### MsABI5 suppresses *MsSMXL1* transcription to positively regulate drought tolerance in apple

SMXL is the key transcriptional repressor regulating SLs-related responses [52, 55]. We identified 19 *SMXL* genes in the apple genome using the GDR and TAIR databases; these genes encode proteins containing Clp conserved domains (Fig. S4, see online supplementary material). To investigate whether SMXLs are involved in the responses of apple to osmotic stress, we measured the expression of these genes in *M. sieversii* plants subjected to osmotic stress. The expression of *MsSMXL1/3/8/15/16* was significantly repressed by osmotic stress (Fig. S5, see online supplementary material). Having demonstrated that SLs-mediated drought resistance in apple functions in an MsABI5-related manner, we investigated whether these *SMXL* genes are regulated by MsABI5. Analysis of the *MsSMXL1/3/8/15/16* promoters identified multiple ACGT or ACE elements, which are involved in ABA responses (Fig. S6A and B, see online supplementary material).

**Figure 4:**
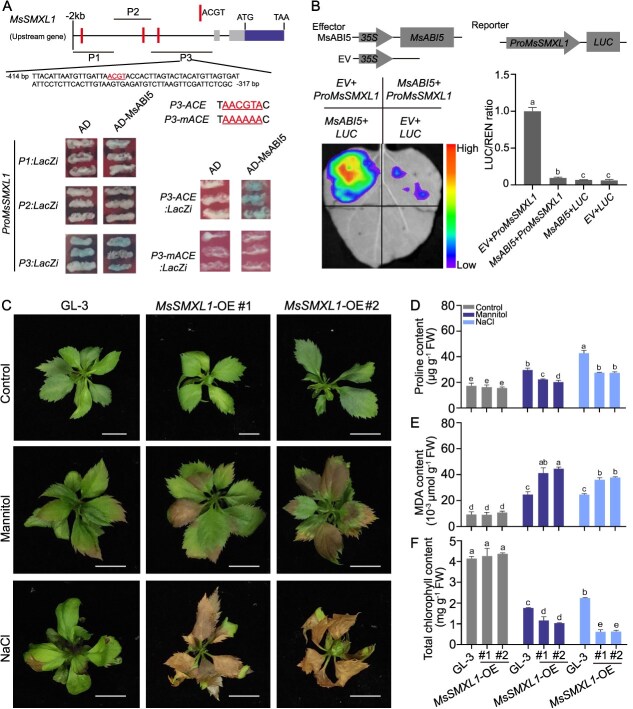
MsABI5 inhibits *MsSMXL1* expression by directly binding to its promoter to improve osmotic stress resistance in apple. A MsABI5 binds to the *MsSMXL1* promoter. Y1H assays were conducted by co-transforming yeast cells with a plasmid expressing *MsABI5* and a plasmid driven by the fragments from the *MsSMXL1* promoter (P1–P3). The empty pB42AD vector (AD) and a plasmid containing the *MsSMXL1* promoter were used as controls. P3-ACE is the ACGT element in P3 fragment of *MsSMXL1* promoter, which is recognized by ABI5. P3-mACE, the ACGT element is mutated from TAACGTAC to TAAAAAAC in P3 fragment of *MsSMXL1* promoter. The promoter fragments containing P3-ACE or P3-mACE were cloned into pLacZi vector. B MsABI5 negatively regulates *MsSMXL1* promoter activity. MsABI5 effector and *MsSMXL1* promoter-LUC reporter plasmids were co-expressed in *N. benthamiana* leaves to analyze LUC activity. EV, empty vector. Three independent infection replicates were performed. Values represent means ± SD. Different letters (a-c) indicate significant differences as determined by LSD range test (*P* < 0.05). C Apple plants overexpressing *MsSMXL1* (*MsSMXL1*-OE) and ‘GL-3’ plants were treated with mannitol or NaCl to induce osmotic stress for 14 days. Drought sensitivity was compared among these apple plants (phenotype). Untreated plants were used as controls. Scale bars, 1 cm. D-F Proline, MDA, and chlorophyll contents were measured in *MsSMXL1*-OE and ‘GL-3’ plants. FW, fresh weight. Nine apple plants were used per treatment, three apple plants were used as one biological replicate, three biological replicates were performed for each treatment, and one representative apple plant for each treatment is pictured. Three independent measurements of proline, MDA, and chlorophyll contents were performed from three biological replicates. Values represent means ± SD. Different letters (a-e) indicate significant differences as determined by LSD range test (*P* < 0.05).

We explored the interactions between MsABI5 and the *MsSMXL1/3/8/15/16* promoters using Y1H assays. MsABI5 bound to the promoter of *MsSMXL1* but not to the promoters of *MsSMXL3/8/15/16* (Fig. S7A, see online supplementary material). We divided the *MsSMXL1* promoter into three fragments (P1–P3) and confirmed this binding using Y1H assays and EMSAs. MsABI5 bound to the P3 fragment, but not to the P1 or P2 fragments (Figs 4A, S6C, and S7B, see online supplementary material). In a dual-LUC assay, the presence of MsABI5 significantly repressed the promoter activity of *MsSMXL1* (Fig. 4B). To further test these results, we measured *MdSMXL1* expression in *MsABI5*-OE and *MsABI5-SRDX* apple plants. *MdSMXL1* was downregulated in *MsABI5*-OE apple plants but upregulated in *MsABI5-SRDX* apple plants compared with ‘GL-3’ with or without drought stress (Fig. S8, see online supplementary material). These results indicate that MsABI5 negatively regulates *MsSMXL1* expression.

To explore the roles of MsSMXL1 in drought tolerance in apple, we overexpressed *MsSMXL1* in ‘GL-3’ (Fig. S9, see online supplementary material) and treated these *MsSMXL1*-OE apple plants and ‘GL-3’ control plants with mannitol or NaCl to compare their sensitivities to osmotic stress. Differences among these plants were not observed in the absence of osmotic stress (Fig. 4C); however, the leaf damage caused by drought was more severe in *MsSMXL1*-OE than in ‘GL-3’ plants (Fig. 4C). When we measured proline, MDA, and chlorophyll contents in these apple plants, they were consistent with the phenotypes: in the absence of osmotic stress, all three were similar among all apple plants, but under osmotic stress, proline and chlorophyll levels were lower and MDA levels were higher in *MsSMXL1*-OE compared with ‘GL-3’ (Fig. 4D–F), indicating a reduction in stress resistance. These results suggest that MsSMXL1 negatively regulates drought tolerance in apple and that MsABI5 suppresses *MsSMXL1* expression to improve drought resistance.

### MsSMXL1 interacts with MsNAC022 to negatively regulate drought resistance and *MsNAC022* is upregulated by MsABI5

**Figure 5:**
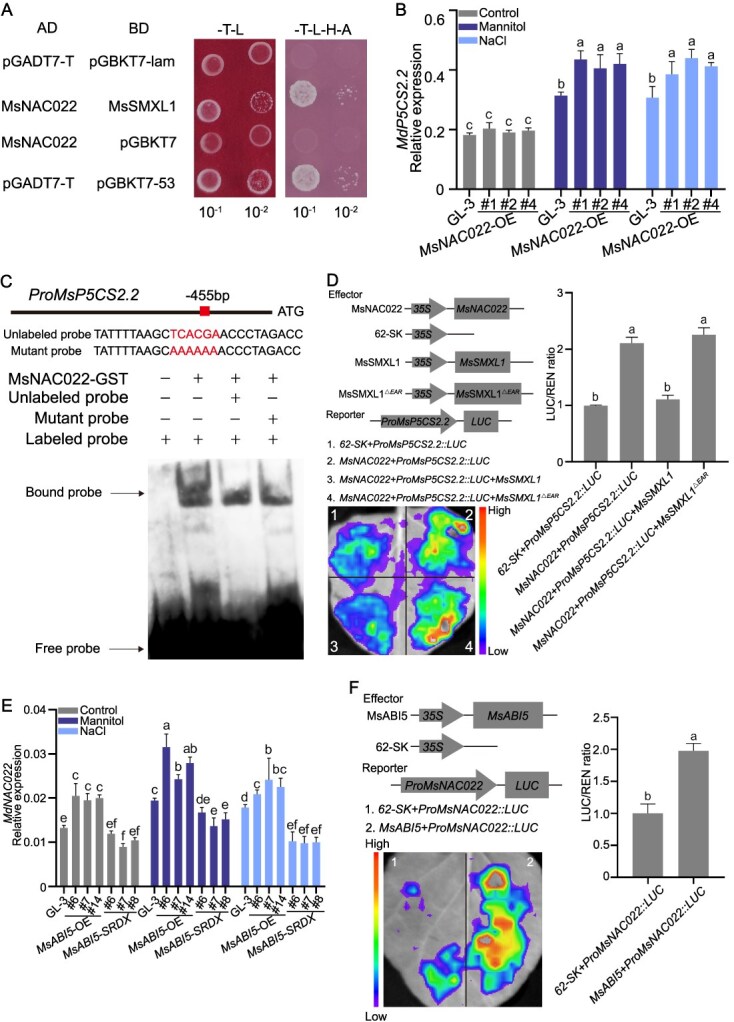
MsSMXL1 interacts with MsNAC022 that is regulated by MsABI5. A Interaction between MsSMXL1 and MsNAC022 analyzed using a yeast two-hybrid assay. Yeast cells were co-transformed with plasmids expressing *MsSMXL1* and *MsNAC022*. Yeast cells containing empty pGADT7-T and pGBKT7-lam vector or pGADT7-T and pGBKT7–53 were used as negative and positive controls, respectively. B Apple plants overexpressing *MsNAC022* and ‘GL-3’ plants were treated with mannitol or NaCl to induce osmotic stress for 14 days. *MdP5CS2.2* expression was analyzed using RT-qPCR. Untreated plants were used as controls. Nine apple plants were used per treatment, three apple plants were used as one biological replicate, and three biological replicates were performed for each treatment. Three independent RNA extractions were performed from three biological replicates. Values represent means ± SD. Different letters (a-c) indicate significant differences by LSD range test (*P* < 0.05). C MsNAC022 binds to the *MsP5CS2.2* promoter. EMSAs were performed with a biotin-labeled *MsP5CS2.2* promoter fragment containing a TCACGA motif. An unlabeled version of the same *MsP5CS2.2* promoter was used as an unlabeled competitor. In the mutant probe, the TCACGA motif was mutated to AAAAAA and used as an unlabeled competitor. D MsNAC022 positively regulates *MsP5CS2.2* promoter activity. The MsNAC022 and MdSMXL1 effectors or the MsNAC022 and MdSMXL1^△EAR^ effectors were co-expressed with the *MsP5CS2.2* promoter-LUC reporter in *N. benthamiana* leaves to analyze LUC activity. The 62-SK empty vector was used as a negative control. Three independent replicates were performed. Values represent means ± SD. Different letters (a and b) indicate significant differences by LSD range test (*P* < 0.05). E *MdNAC022* expression in *MsABI5*-OE and *MsABI5-SRDX* transgenic apple plants and in ‘GL-3’ plants treated with mannitol or NaCl to induce osmotic stress for 14 days, as analyzed using RT-qPCR. Untreated plants were used as controls. The biological replicates were set up the same as Fig. 5B. Values represent means ± SD. Different letters (a-f) indicate significant differences by LSD range test (*P* < 0.05). F MsABI5 positively regulates *MsNAC022* promoter activity. MsABI5 effector and *MsNAC022* promoter-LUC reporter plasmids were co-expressed in *N. benthamiana* leaves to analyze LUC activity. Three independent replicates were performed. Values represent means ± SD. Different letters (a and b) indicate significant differences by LSD range test (*P* < 0.05).

SMXL protein can be degraded by SCF^MAX2^ type E3 ubiquitin ligases mediated by SLs [13]. In this study, we found that the stability of His-tagged MsSMXL1 protein decrease when total protein was added into fusion protein of MsSMXL1-His and MsMAX2-GST, whereas the *rac*-GR24 treatment apparently accelerate the degradation MsSMXL1-His protein (Fig. S16, see online supplementary material). These results indicate that SLs treatment could accelerate degradation of MsSMXL1 protein in MAX2-dependent manner. SMXL transcriptional repressors interact with TFs to inhibit their transcription activation activity and regulate SLs-associated effects [26]. Yeast two-hybrid (Y2H) assays did not reveal a direct interaction between MsSMXL1 and MsABI5 (Fig. S10, see online supplementary material), suggesting that MsSMXL1 might instead interact with TFs downstream of MsABI5. We, therefore, screened for its interacting TFs in a yeast library using MsSMXL1 as bait. MsSMXL1 interacted MsNAC022, which was confirmed by Y2H assay (Fig. 5A). Moreover, pull-down assays verified that MsNAC022-GST can successfully pull down MsSMXL1-His, confirming a direct interaction (Fig. S11A, see online supplementary material).

We previously reported that MsNAC022 enhances drought resistance in apple by promoting ROS scavenging during drought stress [56]. To further investigate its role in drought resistance, we overexpressed *MsNAC022* in ‘GL-3’ and treated these *MsNAC022*-OE apple plants and ‘GL-3’ plants with mannitol or NaCl to induce osmotic stress. All apple plants showed similar performance without drought stress, but overexpressing *MsNAC022* enhanced the osmotic resistance of the apple plants (Fig. S12A, see online supplementary material). We observed higher proline and chlorophyll levels and lower MDA levels during drought stress in *MsNAC022*-OE apple plants than in ‘GL-3’ plants (Fig. S12B−D, see online supplementary material). Coinciding with the enhanced proline accumulation, *MdP5CS2.2* was upregulated in the *MsNAC022*-OE lines compared with ‘GL-3’ (Fig. 5B).

To explore whether MsNAC022 regulates *MsP5CS2.2* expression, we performed Y1H assays, EMSA, and dual-LUC assays. In these assays, MsNAC022 directly bound to the *MsP5CS2.2* promoter and upregulated its expression (Figs 5C and D and S11B, see online supplementary material). SMXL proteins inhibit the transcriptional activities of their interacting TFs through their EAR motifs [13, 57]. We identified this domain in the C-terminus of MsSMXL1 and deleted it to generate the MsSMXL1^△EAR^ protein (Fig. S13, see online supplementary material). To investigate how MsSMXL1 influences the transactivation activity of MsNAC022, we co-expressed the genes encoding MsNAC022 and MsSMXL1 or MsNAC022 and MsSMXL1^△EAR^ with *LUC* driven by the *MsP5CS2.2* promoter in *N. benthamiana* leaves. The transactivation activity of MsNAC022 was significantly repressed by the presence of MsSMXL1, and this repression was abolished when the EAR motif in MsSMXL1 was removed (Fig. 5D). These results indicate that MsNAC022 positively regulates drought resistance, and that MsSMXL1 decreases drought resistance in apple by repressing the transactivation activity of MsNAC022.

To test whether MsNAC022 functions downstream of MsABI5, we examined *MdNAC022* expression in *MsABI5*-OE or *MsABI5-SRDX* apple plants treated with mannitol or NaCl. *MdNAC022* was upregulated in *MsABI5*-OE apple plants and downregulated in *MsABI5-SRDX* apple plants (Fig. 5E). To investigate whether MsABI5 directly regulates *MsNAC022* expression, we performed dual-LUC assays, EMSA, and Y1H assays, finding that MsABI5 positively regulates *MsNAC022* expression by directly binding to its promoter (Figs 5F and S11C and D, see online supplementary material). These results suggest that MsABI5 positively regulates drought resistance by upregulating *MsNAC022*.

## Discussion

### Mechanisms of SL-enhanced drought resistance in apple plants

SLs-enhanced drought resistance is linked to an increase in plant sensitivity to ABA [24, 25, 58]. In this study, *MsABI5*, a key TF in ABA signaling, was induced by SLs during drought stress, and SLs-enhanced drought resistance in apple plants was related to MsABI5 (Figs 2 and 3). SLs-enhanced drought resistance may be related to ABA in apple plants. The interaction between SLs and ABA in regulating drought tolerance of apple plants needs to be further explored in our next work. SLs can also stimulate marked hydrogen peroxide and nitric oxide production to promote stomatal closure, thereby improving drought tolerance in an ABA-independent manner [26]. In addition, we found that *rac*-GR24 treatment increased the aboveground height and leaf number of apple plants during drought stress (Fig. 1A–D), suggesting that SLs may also promote plant growth to enhance drought tolerance, which is consistent with previous studies [59, 60]. We also found that the over-yellowed leaves at bottom of plants existed in *rac*-GR24 treatment/Drought, which may be related to the leaf senescence induced by *rac*-GR24 during drought stress [61]. SLs can be an endogenous regulator of growth and development from root to shoot in plants including enhancement of plant height and stem thickness related to auxin [62, 63], and leaf senescence related to nutrients relocation for development [64], In this study, the expression of auxin-related genes and leaf senescence-related genes was obviously induced by *rac*-GR24 during drought stress (Fig. S14, see online supplementary material), indicating that SLs may activate auxin metabolism and leaf senescence to maintain the growth or development of apple plant under drought condition. Thus, the mechanisms of SL-enhanced drought resistance may be related to multiple pathways, but one of the new mechanisms we found in current study is the molecular pathway of proline accumulation mediated by MsABI5. Proline accumulation, one of the main physiological indicators, is typically seen in plants response to drought stress [5]. In proline biosynthesis, the synthetase P5CS catalyzes glutamate to pyrroline-5-carboxylate (P5C). P5C reductase (P5CR) subsequently reduces P5C to proline [6]. The *P5CS* gene can be induced by drought stress and regulates proline biosynthesis under drought condition, whereas *P5CR* gene is always linked to the redox status of NADPH and NADP^+^ in plants [3, 65]. Improvement of proline biosynthesis by overexpressing *P5CS* gene enhances drought and salt tolerance in plants, which was not credited only to diminished oxidative damage but can be connected to energy regulation and photosynthesis [66, 67]. In Arabidopsis, *ANAC055* induced *P5CS* expression by directly binding to the promoter of *P5CS*, thereby improving proline-mediated drought tolerance [68]. In current study, we found *MsP5CS2.2* had higher expression under osmotic stress (Fig. S2, see online supplementary material). MsABI5 and MsNAC022 both positively regulated *MsP5CS2.2* expression by binding to *MsP5CS2.2* promoter, and promoted proline accumulation, thereby enhancing proline-mediated osmotic stress resistance in apple plants (Figs 2 and 5B–D and S3 and S11B, see online supplementary material). In our research, we have detected the expression of ABA biosynthesis-related genes and ABA catabolism-related genes (identified from GDR database and TAIR database), and found that *rac*-GR24 can repress *MsNCED3.1/3.2* expression but promote the expression of *MsCYP707A1/2* under drought condition. With the concentration of *rac*-GR24 increased, the expression of *MsNCED3.1/3.2* gradually increased, while the expression level of *MsCYP707A1/2* gradually decreased (Fig. S17 see online supplementary material), indicating that other regulatory mechanisms may exist in regulation that SLs repress ABA biosynthesis.

### MsABI5 and MsSMXL1 function in apple plants response to drought tolerance

ABI5, and especially in its role in drought resistance, has been well studied in many plant species, such as Arabidopsis, *Medicago* (*Medicago truncatula*), and apple [48, 50, 69]. We observed that *MsABI5* expression was promoted in apple by drought stress. However, treatment with *rac*-GR24 alone failed to induce *MsABI5* expression unless the plants were under drought stress, indicating that drought stress is important for SLs-mediated induction of *MsABI5* expression (Fig. 2C). Overexpressing *MsABI5* significantly enhanced drought tolerance in apple plants (Fig. 2D–G), which is consistent with previous studies [50]. When the function of MsABI5 was inhibited, *rac*-GR24 treatment failed to improve osmotic tolerance (Fig. 3). These results suggest that SLs-mediated drought resistance in apple is related to MsABI5 and that ABI5 plays an important role in drought resistance, not only in ABA-mediated processes but also in SLs-mediated processes. In our research, the expressions of salt stress response genes (*MsSOS1* and *MsNHX1*) were rapidly induced by *MsABI5* overexpression under NaCl stress, but that was not significantly under mannitol stress (Fig. S15, see online supplementary material). Furthermore, the expressions of drought response genes (*MsRD29A* and *MsRD22*) were obviously activated by MsABI5 under mannitol stress but not NaCl stress, indicating that MsABI5 may play important roles not only in drought response but also in salt stress response.

SMXLs negatively regulate drought resistance in plants. Arabidopsis *smxl6/7/8* mutants exhibit greater drought tolerance than the wild type due to enhanced stomatal closure [70–72]. In this study, we observed that overexpressing *MsSMXL1* led to significantly enhanced sensitivity to osmotic stress in apple plants (Fig. 4C–F), which is consistent with previous studies. MsABI5 directly bound to MsSMXL1 promoter to inhibit MsSMXL1 expression (Figs 4A and B, S7, and S8, see online supplementary material), thereby improving drought resistance, which may be the mechanisms of MsABI5 regulating drought resistance in SLs-mediated processes. SMXLs bind to TFs to repress their activities, regulating the SL responses of plants [73, 74]. In a Y2H assay, MsSMXL1 did not interact with MsABI5 (Fig. S10, see online supplementary material), but it interacted with MsNAC022, which functions downstream of MsABI5 (Figs 5A and S11A, see online supplementary material). The expression of *MsSMXL1* is negatively regulated by MsABI5 (Fig. S8, see online supplementary material). Our results indicate that SLs improve drought resistance in apple by enhancing MsABI5-associated downstream responses rather than by activating MsABI5 itself. Karrikins (KARs), found in smoke and biochar, share closely related perception mechanisms with SLs [75]. They also regulate plant development and abiotic stress responses through KARRIKIN INSENSITIVE2 (KAI2; the receptor for KARs) mediated signaling pathways, which also depend on MAX2-mediated degradation of SMXLs [76]. In Arabidopsis, the activated KAI2 interacts with MAX2 and SMAX1/SMXL2, which leads to the degradation of SMAX1 and SMXL2 [77]. In contrast, the SMXL6/7/8 were degraded by MAX2 in SL signaling pathway of Arabidopsis [13]. In this study, we found that MdSMXL1 and AtSMXL4/5 are clustered together (Fig. S4B, see online supplementary material). The possibility of an interconnection between the SL/KAR signaling pathways and whether MsSMXL1 is degraded through the KAR-mediated signaling pathway remains to be further explored. In plants, ABI5 is a bZIP TF, which is involved in regulating multiple physiological process such as seed germination and responses to drought stress [78]. For example, ABI5 not only acts as an activator for *LEA*, *EM6* and *SBP65* and *SIP1*, but also a suppressor for photosynthesis associated nuclear genes in seed maturation of legumes [79]. In this research, MsABI5 acts as an activator for *MsP5CS2.2* to regulate proline accumulation, and acts as a suppressor for *MsSMXL1* to regulate SL signaling, thereby leading to enhancement of apple drought resistance. In addition, enhancers in downstream genes promoter make TFs activate downstream gene expression, but silencers in promoter of downstream gene can provide the specific binding sites of TF, thereby leading to the case that TF server as the transcriptional repressors [80]. In this study, the enhancers and silencers may exist in *MsP5CS2.2* promoter and *MsSMXL1* promoter, respectively, which may lead to the case that MsABI5 acts as an activator for *MsP5CS2.2* and a suppressor for *MsSMXL1*.

### MsNAC022 and MsSMXL1 are important regulatory nodes downstream of MsABI5 and SLs to cooperatively promote drought resistance in apple plants

NAC TFs are linked to multiple stress responses, such as responses to pathogens, cold stress, alkaline stress, and drought stress [81]. In rice, MNAC3 activates the transcription of immune-suppressive genes to negatively regulate plant immunity to leaf blight diseases [82]. In apple, MdNAC104 directly binds to the *MdCBF1* and *MdCBF3* promoters to positively regulate their expression, thus improving cold tolerance [83]. MdNAC104 negatively regulates alkaline resistance by suppressing γ-aminobutyric acid (GABA) biosynthesis [84]. In rose (*Rosa chinensis*), RcNAC72 interacts with RcDREB2A to improve drought tolerance by promoting *RcRD29A* and *RcRD20* expression [85]. We previously reported that MsNAC022 promotes *MsPOD* expression and the accumulation of peroxidase (POD), which enhances ROS scavenging during drought stress, thereby improving drought resistance in apple [56]. In the current study, we determined that *MsNAC022* is upregulated by MsABI5 and directly promotes proline biosynthesis and drought resistance (Figs 5E and F and S11C and D, see online supplementary material). These findings indicate that MsNAC022 fights drought stress by multiple means. Moreover, MsNAC022 interacted with MsSMXL1, which suppressed its transactivation activity, and this repression was dismissed when the EAR motif was removed from MsSMXL1 (Fig. 5D). These findings indicate that the EAR motif is indispensable for the transcriptional repression activity of MsSMXL1 and that MsSMXL1 functions with MsNAC022 as an important regulatory node downstream of MsABI5 to promote drought resistance in apple. We suggest that this node ensures the direct role of MsABI5 in regulating proline-mediated drought resistance and plays an important supplementary role to MsABI5-mediated drought resistance, which may further intensify ABA signaling to help increase sensitivity to ABA in apple during drought stress, therefore enhancing drought resistance. In addition, SMXL protein with EAR motif can interact and inhibit the TF by itself or cooperating with transcriptional corepressor proteins TOPLESS (TPL) [18, 29, 73, 74, 86]. In Arabidopsis, SMXL6 plus TPL can function as a repressive TF to suppress *SMXL6* expression by directly binding to the promoter of *SMXL6*, thereby forming a negative feedback loop [13]. Furthermore, SMXL protein can also interact with unknown TFs to form a complex that directly regulates the expression of *BRC1* (shoot branching), *TCP1* (leaf elongation) and *PAP1* (anthocyanin biosynthesis) by binding to their promoters [13]. The mechanism whether MsSMXL1 suppresses MsNAC022 by cooperating with TPL or other regulatory modules remains to be explored in the future research.

**Figure 6:**
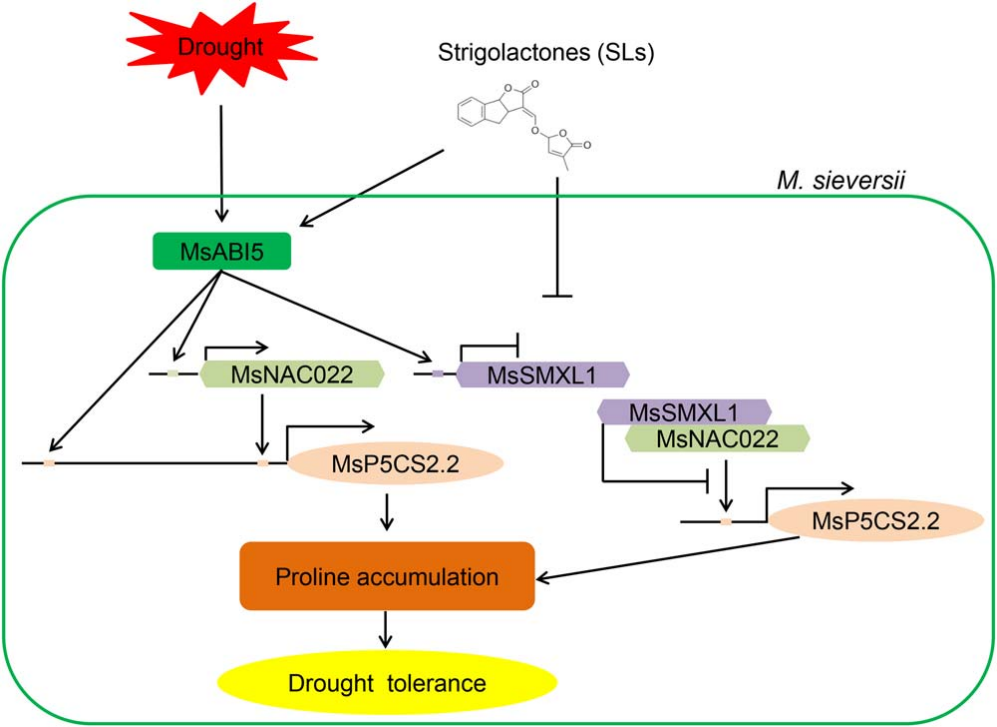
Proposed model for the role of the MsABI5-MsSMXL1-MsNAC022 cascade in enhancing drought resistance. SLs activate *MsABI5* expression during drought stress in *Malus sieversii*. MsABI5 directly binds to the *MsP5CS2.2* promoter and enhances its expression to stimulate proline accumulation and drought resistance. MsABI5 upregulates *MsNAC022* expression but negatively regulates *MsSMXL1* expression. MsNAC022 directly binds to the *MsP5CS2.2* promoter to enhance proline accumulation during drought stress, and MsSMXL1 interacts with MsNAC022 to repress its transactivation activity, thus attenuating drought tolerance in apple. The → indicates activation. The $\perp$ indicates repression. MsABI5 → MsP5CS2.2 indicates *MsP5CS2.2* is activated by MsABI5. MsABI5 → MsNAC022 indicates *MsNAC022* is activated by MsABI5. MsNAC022 → MsP5CS2.2 indicates *MsP5CS2.2* is activated by MsNAC022. MsABI5 $\perp$ MsSMXL1 indicates *MsSMXL1* is repressed by MsABI5. MsSMXL1 $\perp$ MsNAC022 indicates the transactivation of MsNAC022 to *MsP5CS2.2* expression is repressed by MsSMXL1.

Under normal condition, there was no significant difference in *MsP5CS2.2* expression in *MsNAC022*-OE lines and ‘GL-3’ apple plants, but the upregulation of *MsP5CS2.2* expression in *MsNAC022*-OE lines was faster and stronger than that in ‘GL-3’ apple plants under drought condition, thereby leading to more proline accumulation in *MsNAC022*-OE lines during drought stress (Figs 5B and S12B), indicating that drought stress may be important for MsNAC022-mediated induction of *MsP5CS2.2* expression. In addition, MsNAC022 is an NAC TF, which can also be regulated by upstream TFs such as WRKYs, DREBs, and AREBs via stress-related elements of *NAC* promoter and be inhibited by miR164 in post-transcriptional regulation [56, 81]. Similarly, there was no influence on proline content under normal condition in lines of *MsSMXL1*-OE, but proline content in *MsSMXL1*-OE lines increased slower and weaker than that in ‘GL-3’ apple plants under drought condition, indicating that drought may activate proline regulation mediated by MsSMXL1 (Fig. 4D). Furthermore, MsSMXL1 regulated *MsP5CS2.2* expression and proline accumulation through MsNAC022 TF indirectly, indicating that the MsSMXL1-mediated proline accumulation may also affected by other factors interacted with MsSMXL1 such as MAX2 [13] or TFs such as BES1 [87].

Based on our findings, we propose a model in which SLs cooperate with MsABI5 to improve drought resistance in apple (Fig. 6). SLs promote *MsABI5* expression during drought stress. In turn, MsABI5 positively regulates *MsP5CS2.2* expression and proline accumulation to enhance drought tolerance. MsABI5 upregulates *MsNAC022* and downregulates *MsSMXL1* expression. MsNAC022 enhances *MsP5CS2.2* expression and proline accumulation during drought stress, and MsSMXL1 binds to *MsNAC022* and represses its activity, thus attenuating drought resistance in apple. Our work has identified a new molecular module of SLs-enhanced drought tolerance in *M. sieversii*, which may apply to apple breeding to promote proline accumulation during drought stress by activating the MsABI5-MsSMXL1-MsNAC022 module, ultimately enhancing drought tolerance.

## Materials and methods

### Plant material and treatments

The *M. sieversii* and ‘GL-3’ were cultivated on MS medium (Murashige and Skoog medium, catalog no. M0233, Duchefa, The Netherlands) with 0.5 mg L^−1^ IBA (Indole-3-butyric acid, catalog no. 133-32-4, Sigma-Aldrich, USA) and 0.5 mg L^−1^ 6BA (6-Benzylaminopurine, catalog no. 1214-39-7, Sigma-Aldrich, USA) at 23°C, 60% relative humidity, 16 h/8 h (light/dark).

For inducing drought stress to tissue cultured apple plant, the *M. sieversii* plants were cultivated on MS medium containing 1.2 M mannitol (D-mannitol, catalog no. 69-65-8, SCR, Beijing, China) for 14 days, untreated plants were used as controls. Mannitol was used to induce drought stress. Nine apple plants were used per treatment, three apple plants were used as one biological replicate, and three biological replicates were performed for each treatment in this study.

Before natural drought treatment, the *M. sieversii* plants cultivated on MS medium were induced to root with 1/2 MS medium (1/2 Murashige and Skoog medium, catalog no. M0233.0050, Duchefa, The Netherlands) containing 0.5 mg L^−1^ IBA. The rooted *M. sieversii* plants were transferred to soil. For natural drought treatment to soil cultured plants, the soil cultured *M. sieversii* plants with similar growth condition (14–16 leaves, 18–21 cm height) were grown in the plastic pots (22 cm × 22 cm, ~2 L) with the mixture of vermiculite and substrate (Pindstrup Mosebrug, Fabriksvej 2, Denmark) (1:1, v/v), and divided into six groups (three apple plants for each group): Watering normally (Watering); natural drought (Drought); Watering with 5 μM *rac*-GR24 (5 μM *rac*-GR24/Watering); natural drought after 5 μM *rac*-GR24 treatment (5 μM *rac*-GR24/Drought); natural drought after 10 μM *rac*-GR24 treatment (10 μM *rac*-GR24 treatment/Drought); and natural drought after 20 μM *rac*-GR24 treatment (20 μM *rac*-GR24 treatment/Drought). Before treatment, the saturation of soil water content was maintained through irrigation. During drought treatment, the apple plants were grown without water until soil volumetric water content reached 0% for short-term natural drought treatment as previously described [88, 89]. The soil moisture content of apple plants in Watering groups (Watering and 5 μM *rac*-GR24/Watering) are maintained at 40%–45% through daily watering. The soil volumetric water content was measured by soil moisture testing instruments (CP-S, EDKORS, Changzhou, China). For measurement of aboveground height and leaf number of *M. sieversii* plants, three *M. sieversii* plants were used for each treatment. An independent measurement of aboveground height and leaf number of *M. sieversii* plant from each treatment was used as one biological replicate. Three replicates from each treatment were performed. For proline, MDA and chlorophyll determination, three leaves from the top, middle, and bottom of each plant were separately collected excepted for the over-yellowed leaves at bottom of plants in *rac*-GR24 treatment/Drought; a total of 27 leaves were collected from each treatment and evenly divided into three sets named treatment-1, 2, and 3 (e.g. Drought-1, 2, and 3) as three biological replicates per treatment, froze them with liquid nitrogen, and stored at −80°C. For RNA extraction or measurement of physiological indicators (MDA, proline and chlorophyll), the leaves of each biological replicate stored at −80°C were grounded into powder under liquid nitrogen and divided into three sets named treatment 1-1, 2, and 3, treatment 2-1, 2, and 3, treatment 3-1, 2, and 3 (e.g. Drought 1–1, 2, and 3, Drought 2-1, 2, and 3, Drought 3-1, 2, and 3) as three technical replicates performed in the experiment of each biological replicate.

The ‘GL-3’ (*M. domestica*) cultivated on MS medium was used for apple transformation and relative experiments. For inducing drought stress, the MS medium was added with 200 mM NaCl (Sodium chloride, catalog no. 7647-14-5, Tongguang chemical, Beijing, China) or 300 mM mannitol as previously described [90]. Nine apple plants for transgenic lines or ‘GL-3’ were used per treatment, three apple plants were used as one biological replicate, and three biological replicates were performed for each treatment.

For SLs treatment to *MsABI5-SRDX* apple plants, the plants were cultivated on MS medium containing 20 μM *rac*-GR24 with either 300 mM mannitol or 200 mM NaCl for 14 days. Nine apple plants were used per treatment, three apple plants were used as one biological replicate, and three biological replicates were performed for each treatment in this study.

### RNA extraction and RT-qPCR analysis

Total RNA was extracted from apple plants using Plant Total RNA Extract Kit (catalog no. DP441, Tiangen, Beijing, China). The cDNA was generated using HiScript^®^ II 1st Strand cDNA Synthesis Kit (catalog no. R223-01, Vazyme, Nanjing, China). The RT-qPCR was performed in Rotor-Gene Q system (R0913110, QIAGEN Hilden, Germany) with SYBR qPCR Mix (catalog no. ZF502, Zoman, China). *Histone H3* was used as an internal quantification control. Relative expression was calculated using 2^−ΔΔCt^ [91, 92]. Three independent RNA extractions from three biological replicates were performed. The primers are listed in Table S1 (see online supplementary material). The accession number of genes are listed in Table S2 (see online supplementary material).

### Phylogenetic analysis

The SMXLs amino acid sequences from Arabidopsis were used for query sequences Blast P in apple genome (https://www.rosaceae. org/species/malus/malus×domestica/genome GDDH13 v1.1). The functional domains were identified by SMART (http://smart.embl-heidelberg.de/). The phylogenetic trees were constructed by the Mega X software using neighbor-joining calculating model with 1000 bootstrap replicates [93].

### Plasmid constructs and apple genetic transformation

For creating overexpression apple plants, the full-length coding sequence (CDS) from *MsABI5* or *MsSMXL1* without the termination codon were separately inserted into the pCAMBIA1302 vector with a C terminal a GFP tag peptide (pCAMBIA1302-GFP vector). For gene functional inhibition, the sequence from SRDX motif was fused down stream of *MsABI5* CDS, and this fusion gene was ligated into pCAMBIA1302-GFP vector. These plasmids were separately transformed into *Agrobacterium* strain EHA105 for apple genetic transformation. The genetic transformation in ‘GL-3’ with slight modification was performed as described [90]. The young leaves were cut into small pieces and infected by *Agrobacterium* that contained recombinant plasmid for 10 minutes. After incubation, leaves were cultivated on MS medium containing 0.5 mg L^−1^ IBA and 4 mg L^−1^ 6BA at dark for 3 days. Washing the infected leaves with water for three times, and the leaves were continued to cultivate on MS medium containing 0.5 mg L^−1^ IBA, 4 mg L^−1^ 6BA, 400 mg L^−1^ cefotaxime, and 4 mg L^−1^ hygromycin B (REF no. 10843555001, Roche, Germany) for 30 days. When callus was induced on infected leaves, the callus was cultivated under light for inducing the transformation to apple plants. Finally, the induced apple plants were cultivated in 1/2 MS medium containing 0.5 mg L^−1^ IBA to stimulate root growth. The gene expression was analyzed by RT-qPCR to identify whether the apple plant was transformed successfully.

### Yeast one-hybrid assay

The CDS for *MsABI5* and *MsNAC022* were individually inserted into pB42AD vector. Full-length promoters from *MsSMXL1*, *MsNAC022*, *MsP5CS2.2*, and the promoter fragments containing P3-ACE or P3-mACE elements from *MsSMXL1* were separately cloned into the pLacZi vector. P3-ACE is the ACGT element in P3 fragment of MsSMXL1 promoter, which is recognized by ABI5. P3-mACE, the ACGT element is mutated from TAACGTAC to TAAAAAAC in P3 fragment of MsSMXL1 promoter. Y1H assay was performed as described [94].

### Dual-luciferase assay

For effector vector, the CDS from *MsABI5* and *MsNAC022* were separately cloned into pGreenII 0029 62-SK vector. The *MsSMXL1*, *MsNAC022*, and *MsP5CS2.2* promoters were separately ligated into pGreenII 0800-LUC vector to generate reporter vectors. The CDS of *MsSMXL1* without EAR motif (*MsSMXL1^△EAR^*) was cloned into pSPYCE-35S vector. The empty vectors of pGreenII 0029 62-SK and pGreenII 0800-LUC were used as negative controls. These vectors were co-expressed in *N. benthamiana* as described previously [56]. The LUC image was captured by chemiluminescence analysis system (LB985, Berthold, Germany), and LUC/REN ratio were determined using Dual-luciferase Reporter Assay Kit (catalog no. DL101-01, Vazyme, Nanjing, China) following the instructions of manufacturers.

### Electrophoretic mobility-shift assay

The 5′ biotin-labeled probes containing the ACGT motifs from *MsSMXL1, MsNAC022,* or *MsP5CS2.2* promoters, or containing CACG motifs from *MsP5CS2.2* promoter were, respectively, synthesized by DIA-UP Biotech (Beijing, China). The CDS from MsABI5 and MsNAC022 was separately ligated into pMAL-C2X-MBP or pGEX4T-1-GST vector, purification of MsABI5-MBP or MsNAC022-GST protein was performed as described [11]. EMSA was performed as manufacturers’ instructions (Beyotime, Shanghai, China).

### Yeast library screening and Y2H assays

To screen the penitential proteins interacted with MsSMXL1, we constructed the yeast library from *M. sieversii* plant, which was subjected to drought stress. The CDS of *MsSMXL1* was cloned into pGBKT7 vector as the bait. Yeast library screening was performed following the manufacturer’s instructions. The bait plasmid was transformed into yeast strain Y2HGold and cultivated on SD/-Trp/-Leu medium (catalog no. 630417, Takara, Japan). The positive clone was selected by SD /-Trp/-Leu/-His/-Ade medium (catalog no. 630428, Takara, Japan) with X-α-Gal (catalog no. 107021-38-5, LABLEAD, Beijing, China).

The CDS of MsNAC022 was cloned into pGADT7 vector to construct AD-MsNAC022 plasmid. The CDS of MsSMXL1 was introduced into pGBDT7 vector to construct BD-MsSMXL1 plasmid. These plasmids were co-transformed into yeast strain Y2HGold and cultivated on SD/-Trp/-Leu medium (catalog no. 630417, Takara, Japan). The positive clone was identified by SD/-Trp/-Leu/-His/-Ade medium (catalog no. 630428, Takara, Japan). The Y2H assay for MsNAC022 and MsSMXL1 was performed as described [95].

Plasmids for LexA-MsSMXL1 and AD-MsABI5 were co-transformed into the EGY48 plus H18 yeast strain and cultivated on SD/-Trp/-Ura medium (catalog no. PM2260, Coolaber, Beijing, China). The positive clone was identified by SD/-Trp/-Ura/-His medium (catalog no. Y408325, LABLEAD, Beijing, China) with X-Gal (catalog no. X0300, LABLEAD, Beijing, China). The Y2H assay for MsSMXL1 and MsABI5 was performed as described [96].

### Pull-down assay

The CDS of *MsSMXL1* were cloned into pET28A and this protein was purified as described [95]. The MsNAC022-GST fusion protein (20 μg) was conjugated to GST beads (catalog no. 70601, Beaver Bio, Suzhou, China) and then incubated with MsSMXL1-His fusion protein (2 μg) on ice for 1 hour. The beads were washed using PBS (catalog no. P1020, Solarbio Life Science, Beijing, China) for three times. The proteins were collected with 200 μl glutathione elution buffer and analyzed using western immunoblot with His antibody. GST protein was used as a negative control.

### Protein stability assays

The method of protein stability was based on cell-free degradation assays with slightly modifications [94]. MsMAX2-GST and MsSMXL1-His fusion proteins were prepared. Leaves of *M. sieversii* plant was used to extract total protein. The fusion protein, total protein, and *rac*-GR24 were mixed and incubated at 37°C for 2 hour, then which were detected using GST antibody and His antibody to observe protein bands through western blot.

### Determination of proline, MDA, chlorophyll, and ABA

The determination of Proline, MDA, and chlorophyll were performed as previously described [97]. The ABA content was determined using a Plant ABA Elisa Kit (catalog no. YS07115b, YaJi Biological, Shanghai, China) following the manufacturers’ instructions.

### *Cis*-elements identification for *MsSMXLs* promoter

The Plant Genomic DNA Kit (TIANGEN, Beijing, China) was used to extract genomic DNA from *M. sieversii*. The reference sequence of *SMXLs* promoters were selected from GDR database (https://www.rosaceae.org/) and NCBI database (https://www.ncbi.nlm.nih.gov/). The sequence of *SMXLs* promoters was cloned from *M. sieversii* genomic DNA and submitted to the Plant Care database (http://bioinformatics.psb.ugent.be/webtools/plantcare/html/) for *cis*-elements analysis.

### Statistical analysis

Data are presented as means ± SD. Results were analyzed by GraphPad Prism 9.3.1. The statistical analyses between two groups were performed through Student’s *t* test. The least significance difference test was performed for analyzing significant differences among multiple groups.

## Supplementary Material

Web_Material_uhaf101

## Data Availability

The data that support the findings of this study are available from the corresponding author upon reasonable request.
