# Supplementary material for: Strigolactones enhance apple drought resistance via the MsABI5-MsSMXL1-MsNAC022 cascade
Source: Hortic Res. 2025 Apr 9;12(7):uhaf101. doi: 10.1093/hr/uhaf101 (PMC12090352; doi:10.1093/hr/uhaf101)
Supplement: Web_Material_uhaf101 [file web_material_uhaf101.zip › Clean version of the Supplementary Figure-0222.docx]

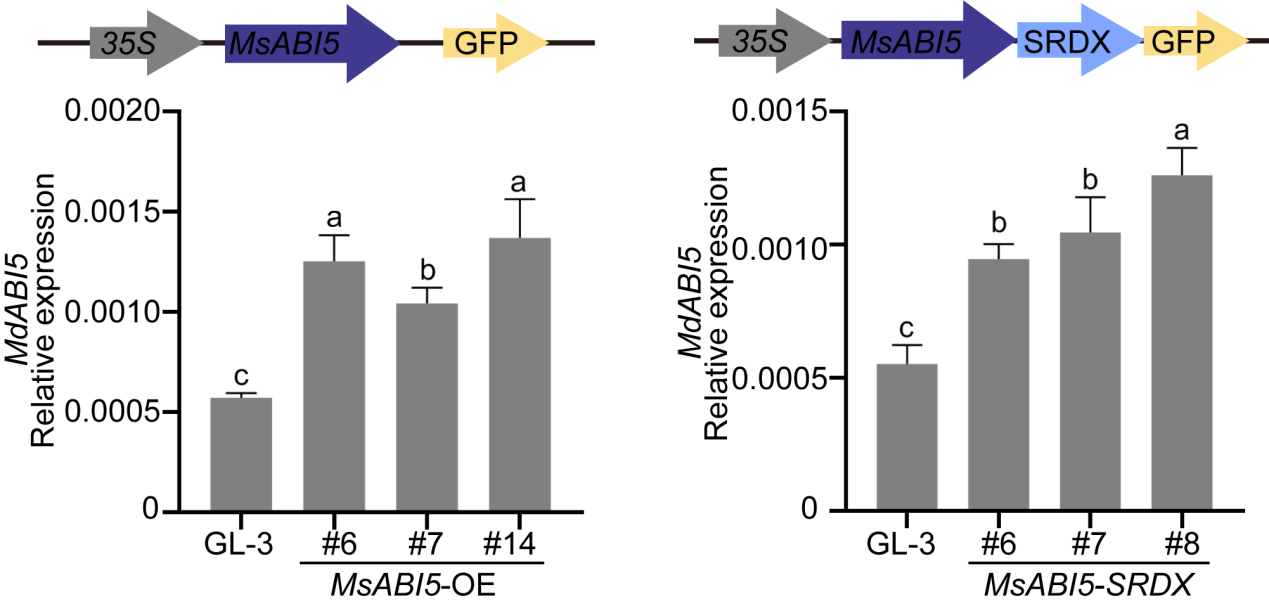


**Figure S1. Identification of *MsABI5-*OE and *MsABI5-SRDX* transgenic apple plant.** Three *MsABI5* overexpression lines (MsABI5-OE) and three lines expressing a repressor version of *MsABI5* (MsABI5-SRDX) were identified using RT-qPCR. Each apple plant from each transgenic line was used as one biological replicate; three replicates were performed for each transgenic line. SRDX, transcriptional repressor domain. Values represent means ± SD. Different letters (a−c) indicate significant differences by LSD range test (*P* < 0.05).


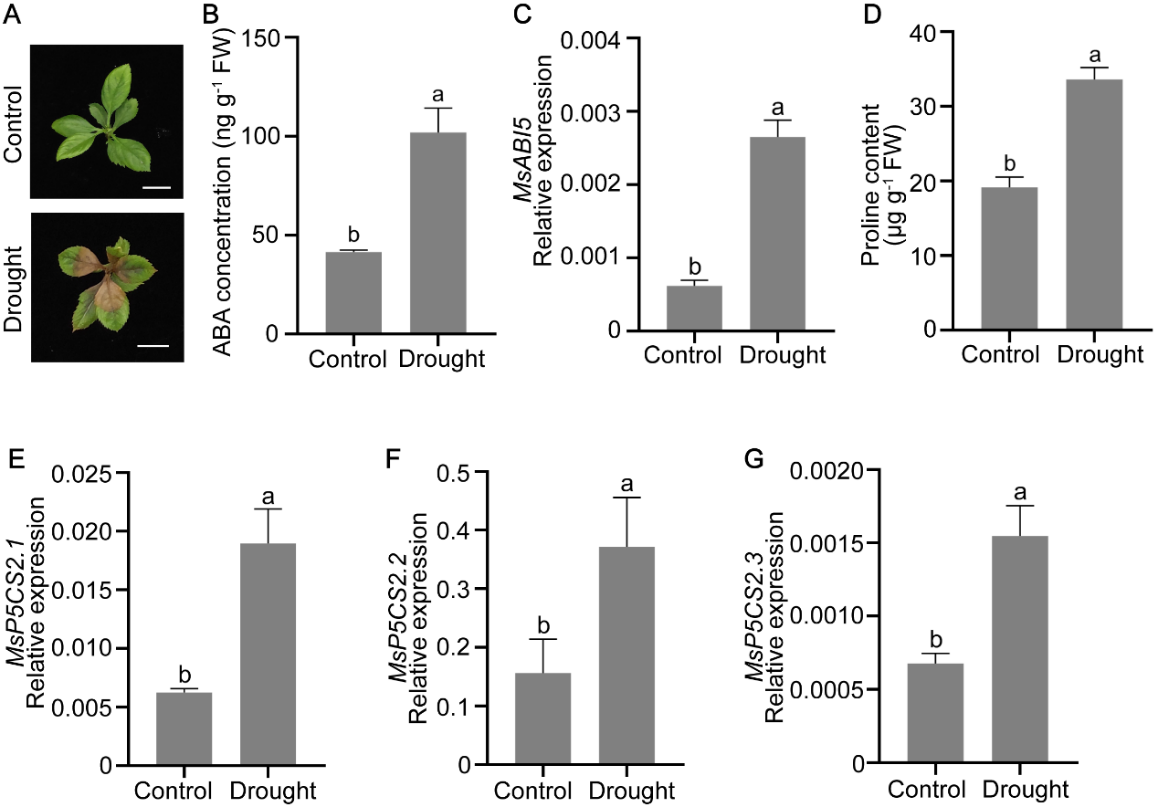


**Figure S2. Responses of *MsABI5* and *MsP5CSs* to drought stress. A** *Malus sieversii* plants were treated with 1.2 M mannitol for 14 d to induce osmotic stress. Control, untreated plants. **B** ABA levels in *Malus sieversii* plants. The apple plants were treated with 1.2 M mannitol for 14 d to induce osmotic stress, and ABA levels were measured. Control, plants treated without mannitol. Drought, mannitol-treated apple plants. *MsABI5* expression **(C)**, proline levels **(D),** and *MsP5CS2.1/2.2/2.3* expression **(E−G)** were measured. Nine apple plants were used per treatment, three apple plants were used as one biological replicate, and three biological replicates were performed for each treatment. Three independent RNA extractions or proline content measurements were performed on three biological replicates. Values represent means ± SD. Different letters (a and b) indicate significant differences by LSD range test (*P* < 0.05).


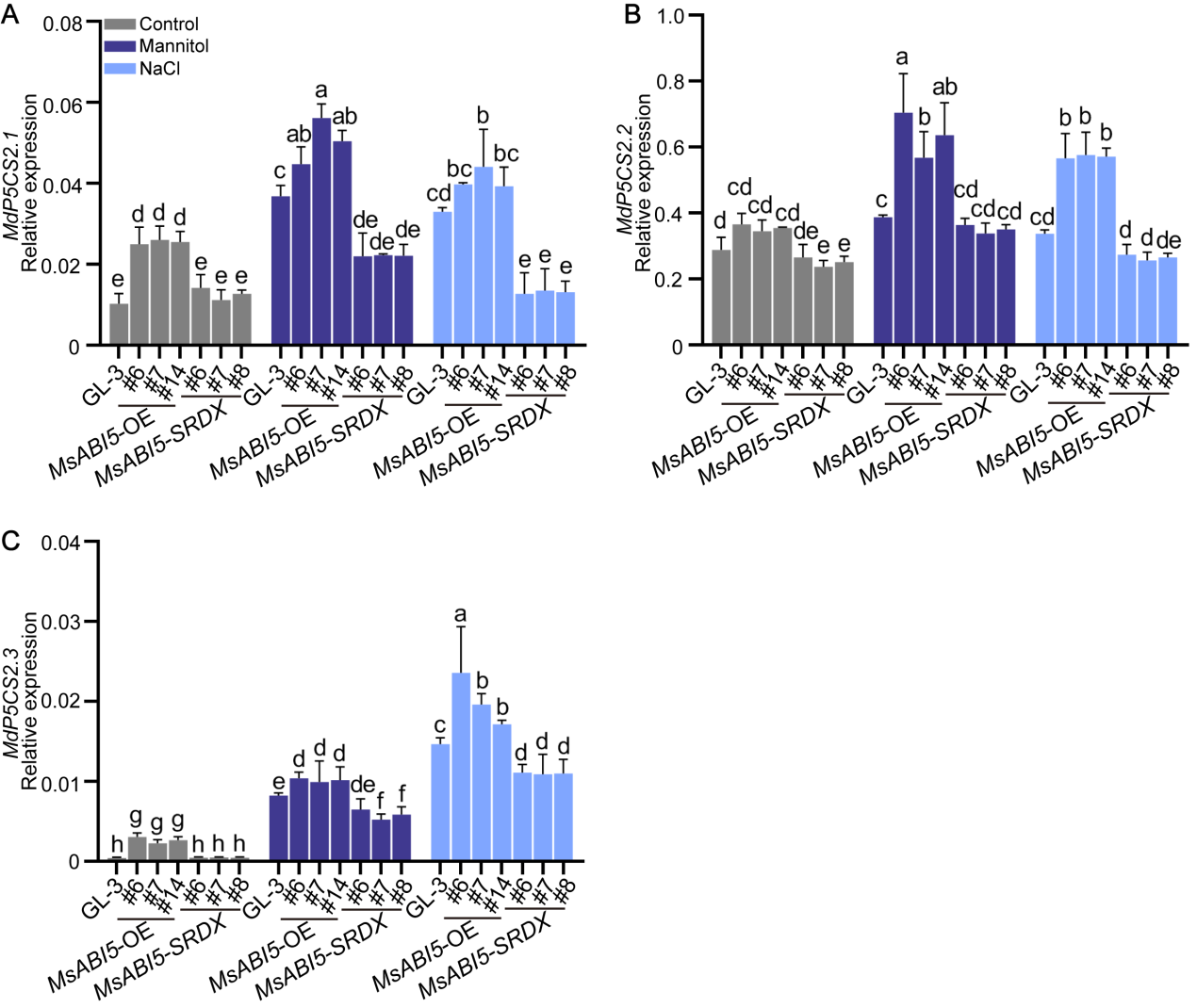


**Figure S3. Expression of *MdP5CSs* in *MsABI5* transgenic apple plants. A−C** Apple plants overexpressing *MsABI5* (MsABI5-OE) or expressing a repressor version of *MsABI5* (MsABI5-SRDX) and ‘GL-3’ plants were treated with mannitol or NaCl to induce osmotic stress for 14 d. Expression of *MdP5CS2.1*, *MdP5CS2.2*, and *MdP5CS2.3* was detected using RT-qPCR. Untreated plants were used as controls. Nine apple plants were used per treatment, three apple plants were used as one biological replicate, and three biological replicates were performed for each treatment. Three independent RNA extractions were performed from three biological replicates. The *x*-axis indicates the transgenic line numbers. Values represent means ± SD. Different letters (a−h) indicate significant differences by LSD range test (*P* < 0.05).


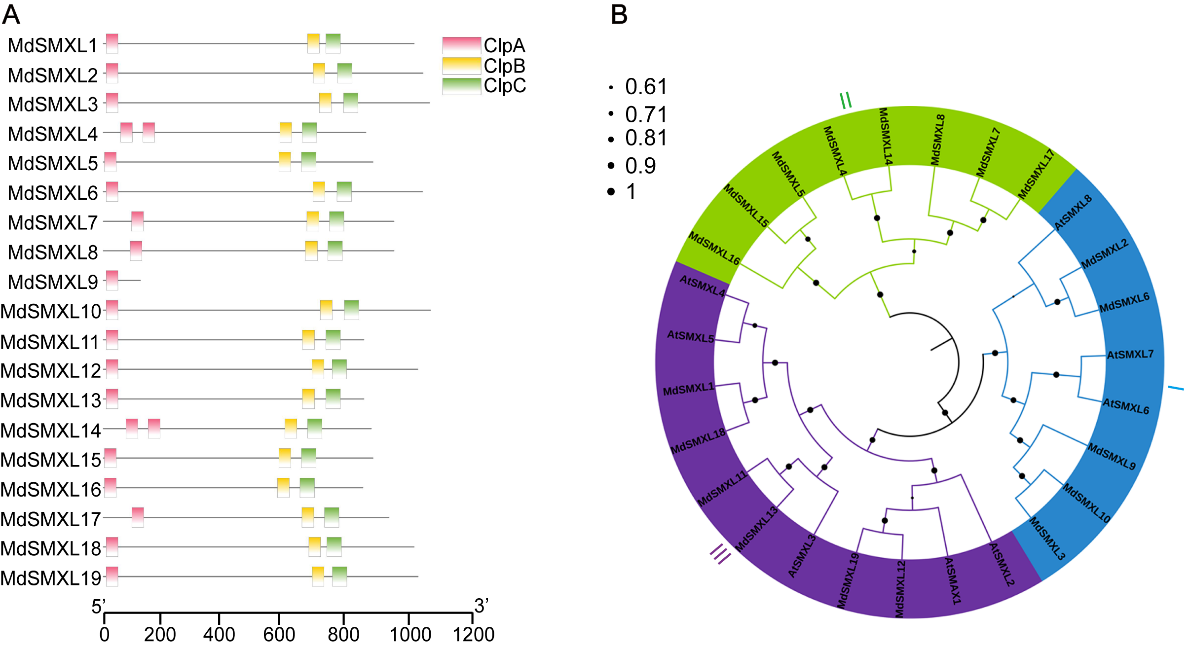


**Figure S4. Identification of *MsSMXL* genes.**

**A** Conserved structural domain analysis. **B** Phylogenetic analysis.


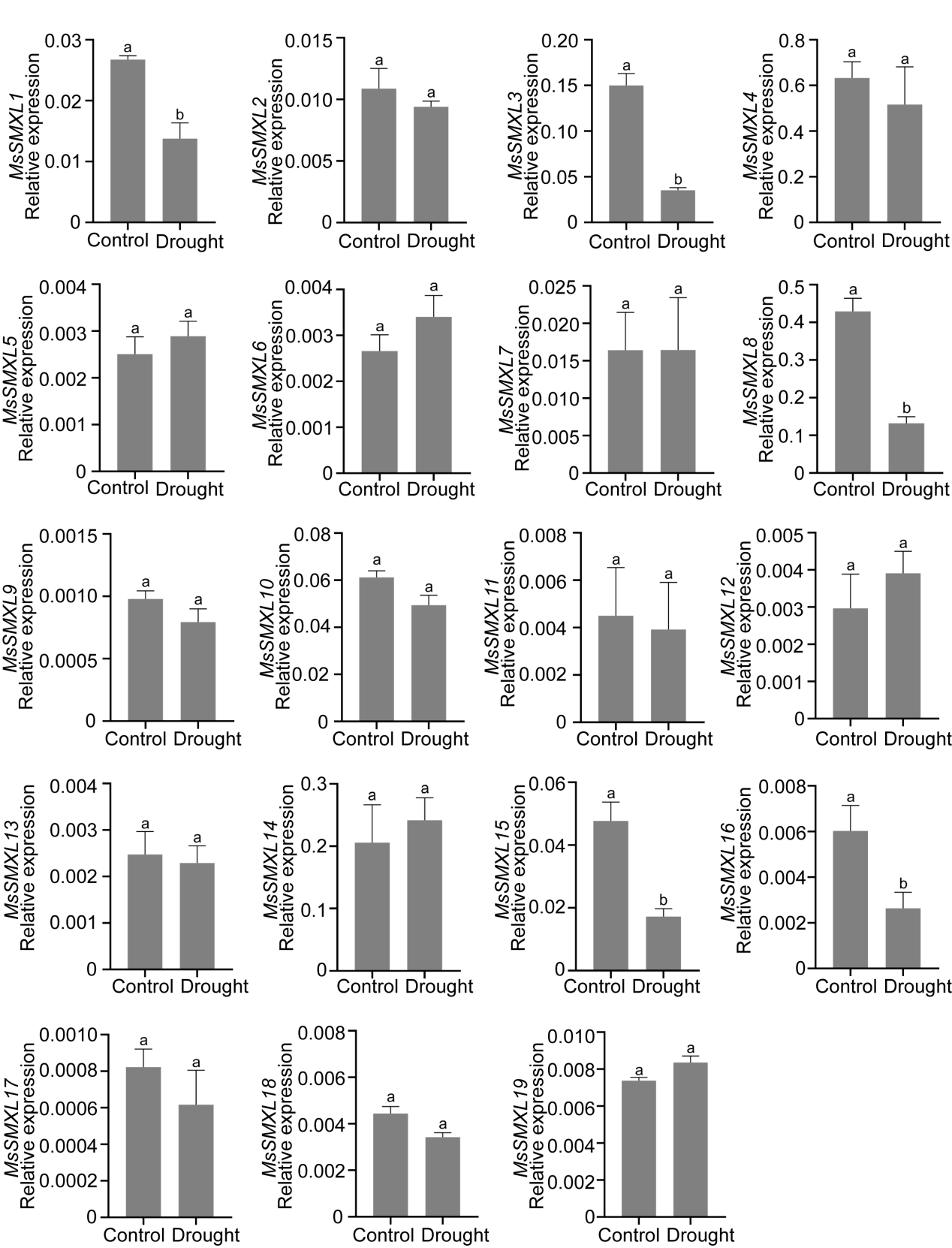


**Figure S5. Expression of *MsSMXL* genes in response to drought stress.** *Malus sieversii* plants were treated with mannitol for 14 d to induce osmotic stress. Control, untreated plants. The expression of *SMXL* genes was detected using RT-qPCR. Nine apple plants were used per treatment, three apple plants were used as one biological replicate, and three biological replicates were performed for each treatment. Three independent RNA extractions were performed from three biological replicates. Values represent means ± SD. Different letters (a and b) indicate significant differences by LSD range test (*P* < 0.05).

**
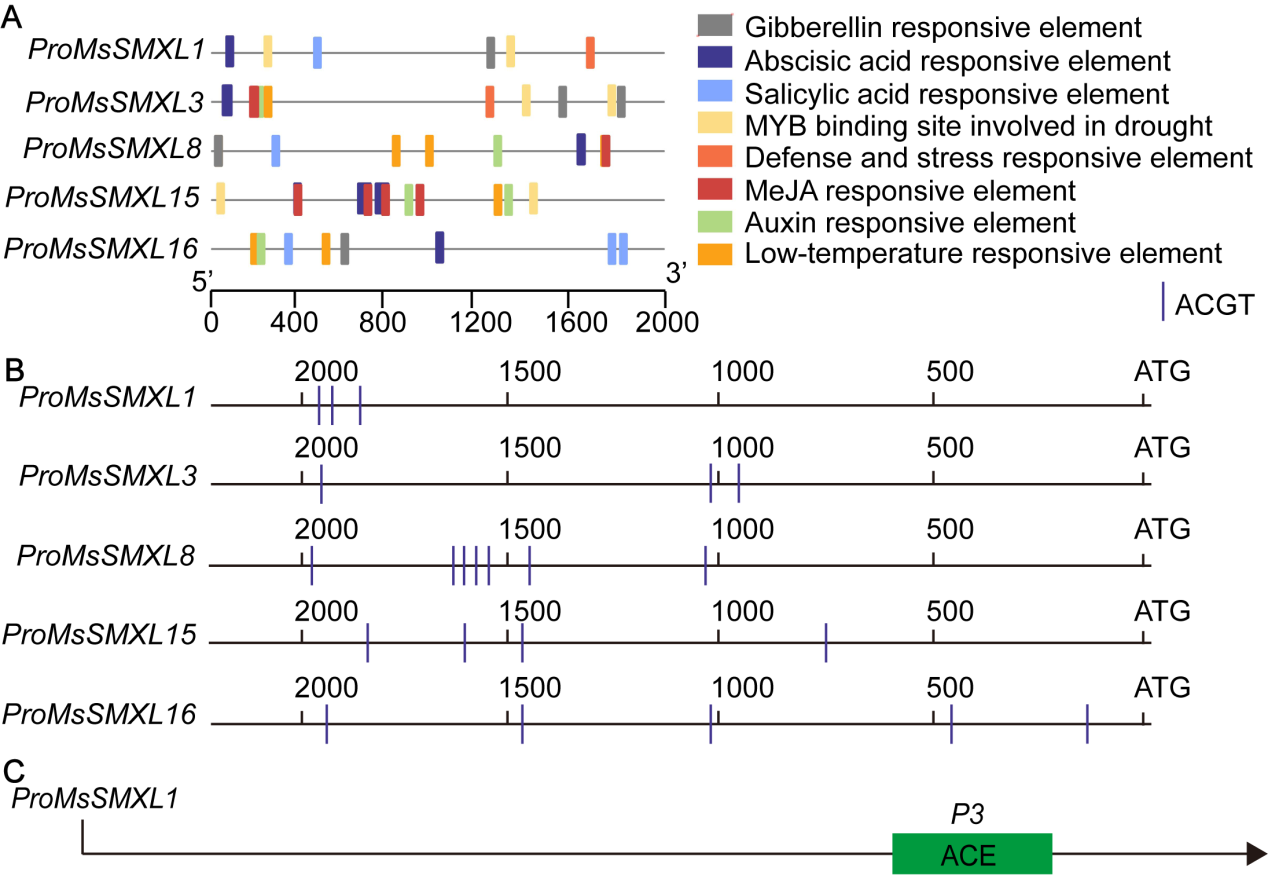
**

**Figure S6. Elements in the *MsSMXL1*, *MsSMXL3*, *MsSMXL8*, *MsSMXL15*, and *MsSMXL16* promoters. A−B** Elements and ACGT motifs identified in the *SMXL* promoters. **C** Model of the P3 fragment of the *MsSMXL1* promoter. ACE represents the ACGT element.


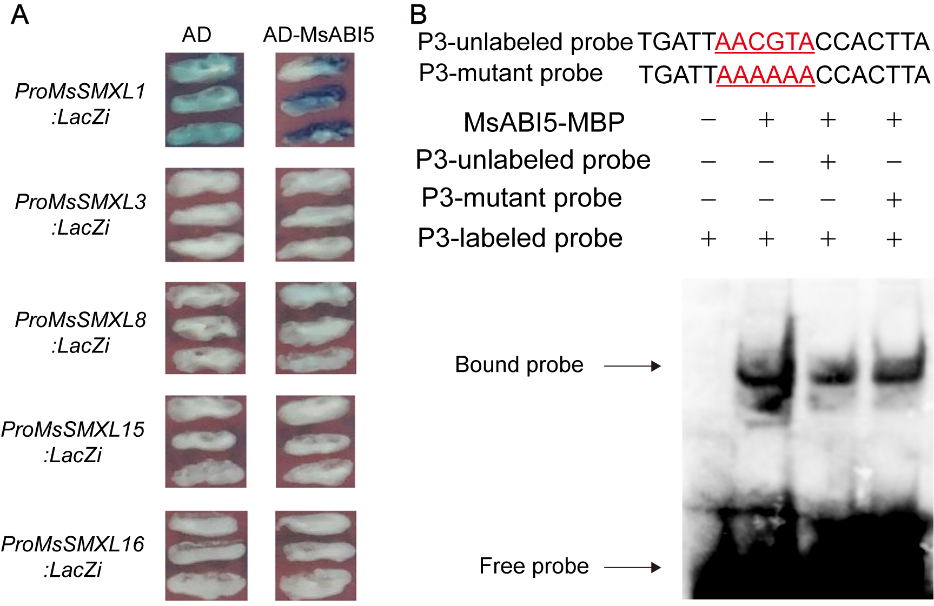


**Figure S7 MsABI5 binds to the *MsSMXL1* promoter. A** Yeast one-hybrid assays were conducted by co-transforming yeast cells with a plasmid expressing *MsABI5* and a plasmid driven by the *MsSMXL1/3/8/15/16* promoters. **B** MsABI5 binds to the ACE element in the *MsSMXL1* promoter. EMSAs were performed with a biotin-labeled *MsSMXL1* promoter fragment containing an ACE element. An unlabeled version of the same *MsSMXL1* promoter was used as an unlabeled competitor, and an unlabeled version of the fragment with the mACE element was used as the mutant probe.

**
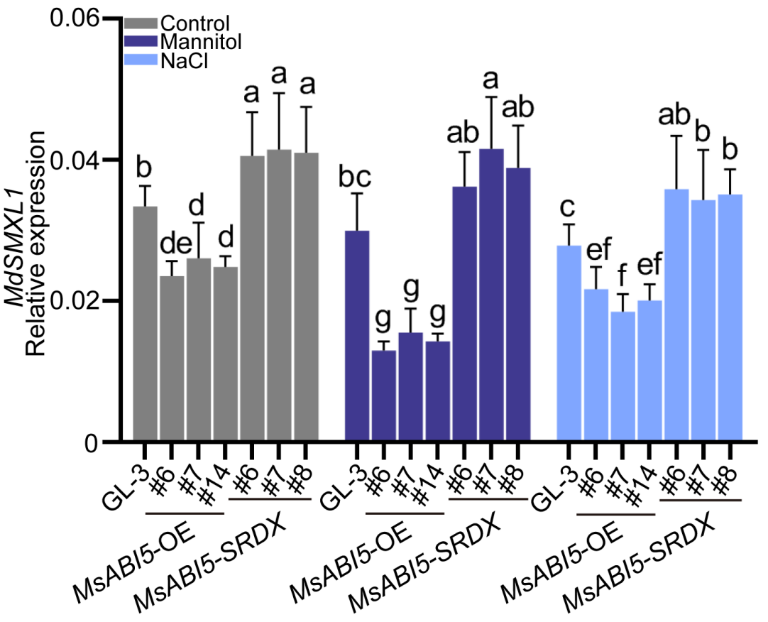
**

**Figure S8. *MdSMXL1* is downregulated by MsABI5.** Apple plants overexpressing *MsABI5* (MsABI5-OE) or expressing a repressor version of *MsABI5* (MsABI5-SRDX) and ‘GL-3’ plants were treated with mannitol or NaCl to induce osmotic stress for 14 d, and the expression of *MdSMXL1* was detected using RT-qPCR. Untreated plants were used as controls. Nine apple plants were used per treatment, three apple plants were used as one biological replicate, and three biological replicates were performed for each treatment. Three independent RNA extractions were performed from three biological replicates. The *x*-axis indicates the transgenic line numbers. Values represent means ± SD. Different letters (a−g) indicate significant differences by LSD range test (*P* < 0.05).


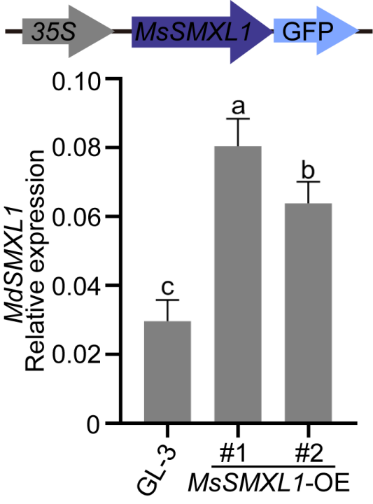


**Figure S9.** **Identification of *MsSMXL1-*OE transgenic apple plants.** Two *MsSMXL1* overexpression lines were identified using RT-qPCR. One apple plant from each transgenic line was used as one biological replicate, and three replicates were performed for each transgenic line. Values represent means ± SD. Different letters (a−c) indicate significant differences by LSD range test (*P* < 0.05).


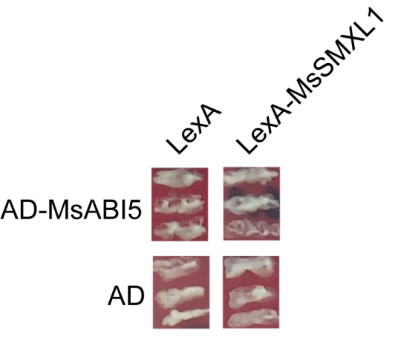


**Figure S10. MsABI5 does not interact with MsSMXL1 in a Y2H assay.** MsABI5 was fused with the activation domain (AD) in pB42AD. MsSMXL1 was fused with the LexA DNA-binding domain in pEG202. Yeast cells were co-transformed with these vectors, and the interactions of the fusion proteins were detected by X-Gal treatment.


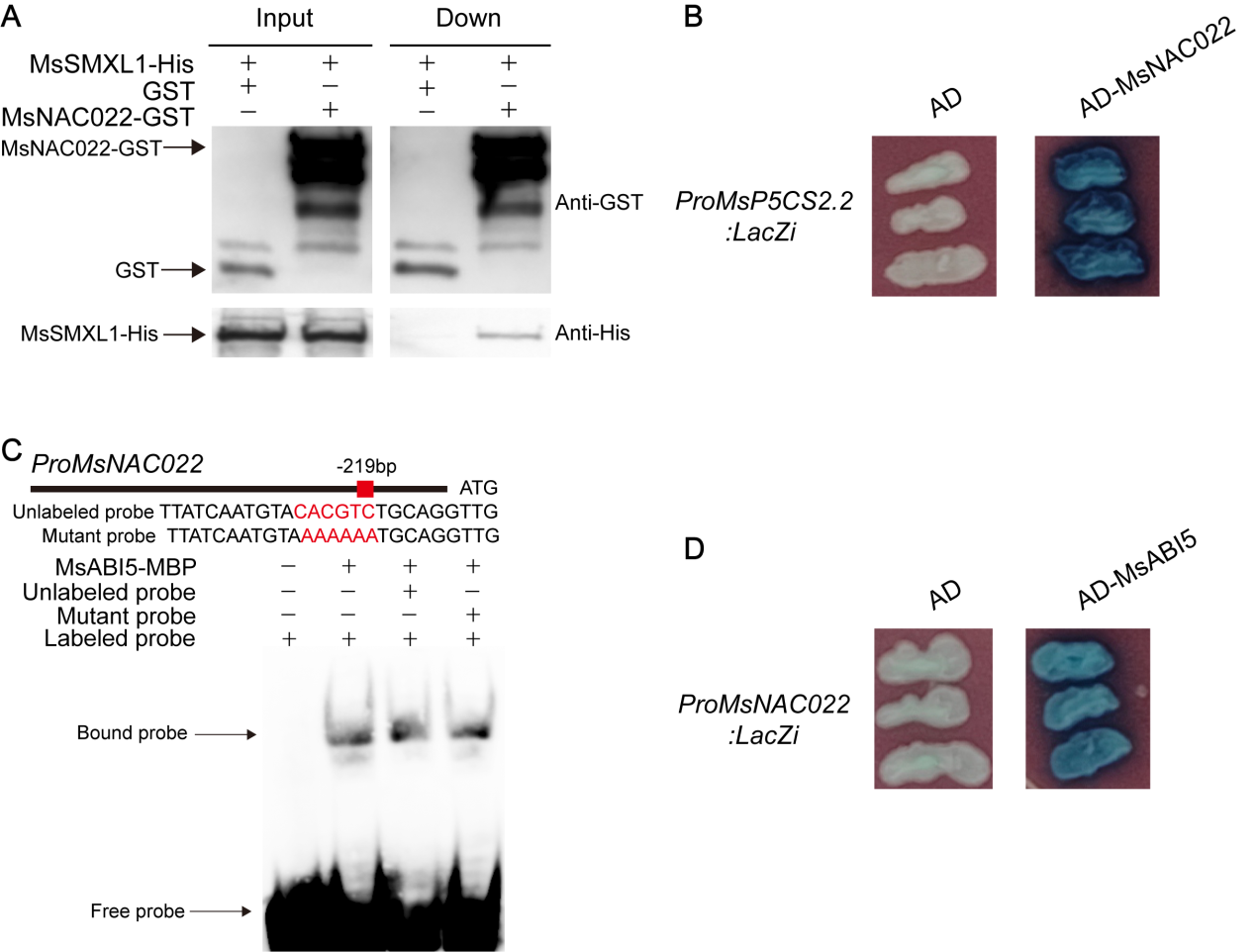


**Figure S11. MsNAC022 interacts with MsSMXL1 and *MsNAC022* is regulated by MsABI5 through the interaction of promoter. A** Analysis of the interaction between MsSMXL1 and MsNAC022 using pull-down assays. MsABI5 was fused to His to yield MsABI5-His. MsNAC022 was fused to GST to yield MsNAC022-GST. These proteins were expressed from pET30a or pGEX4T-1. MsABI5-His was incubated with either GST or MsNAC022-GST, and GST-tagged proteins were pulled down by GST magnetic beads. Anti-GST or anti-His antibodies were used for immunoblotting. Bands detected by anti-His antibody in the pull-down samples indicate that MsSMXL1 interacts with MsNAC022. **B** MsNAC022 binds to the *MsP5CS2.2* promoter. Yeast one-hybrid assays were conducted by co-transforming yeast cells with a plasmid expressing *MsNAC022* and a plasmid driven by the *MsP5CS2.2* promoter. The empty pB42AD vector (AD) and a plasmid containing the *MsP5CS2.2* promoter were used as controls. **C** MsABI5 binds to the *MsNAC022* promoter. EMSAs were conducted with a biotin-labeled *MsNAC022* promoter fragment containing an ABRE. An unlabeled version of the same *MsNAC022* promoter fragment was used as an unlabeled competitor. For the mutant probe, the ABRE was mutated into AAAAAA and used as an unlabeled competitor. **D** MsABI5 binds to the *MsNAC022* promoter. Yeast one-hybrid assays were performed by co-transforming yeast cells with a plasmid containing *MsABI5* and a plasmid driven by the *MsNAC022* promoter. The empty pB42AD vector and a plasmid containing the *MsNAC022* promoter were used as controls.


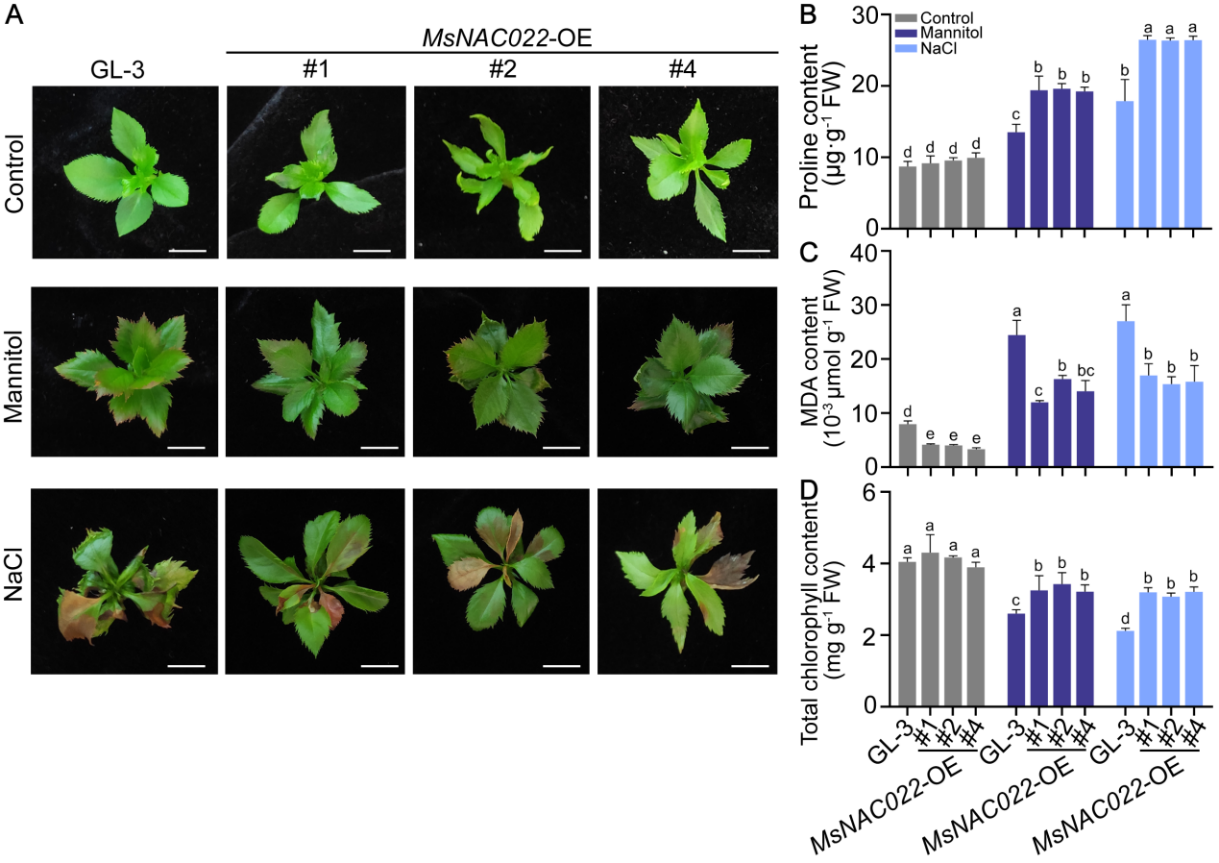


**Figure S12.** **MsNAC022 positively regulates drought tolerance in apple. A** Apple plants overexpressing *MsNAC022* (MsNAC022-OE) and ‘GL-3’ plants were treated with mannitol or NaCl to induce osmotic stress for 14 d. Drought sensitivity was compared among these apple plants (phenotype). Untreated plants were used as controls. Scale bars, 1 cm. **B−D** Proline, MDA, and chlorophyll contents were measured in MsNAC022-OE or ‘GL-3’ plants. Nine apple plants were used per treatment, three apple plants were used as one biological replicate, three biological replicates were performed for each treatment, and one representative apple plant from each treatment is pictured. Three independent measurements of proline, MDA, and chlorophyll contents were performed from three biological replicates. The *x*-axis indicates the transgenic line number. FW, fresh weight. Values represent means ± SD. Different letters (a−e) represent significant differences by LSD range test (*P* < 0.05).


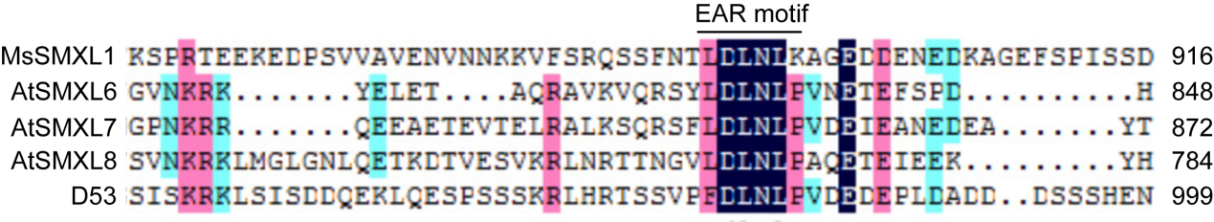


**Figure S13. Amino acid sequence alignment of MsSMXL1.** EAR is a transcriptional repression motif. D53 is a homologous protein of *AtSMXL6/7/8* in rice.


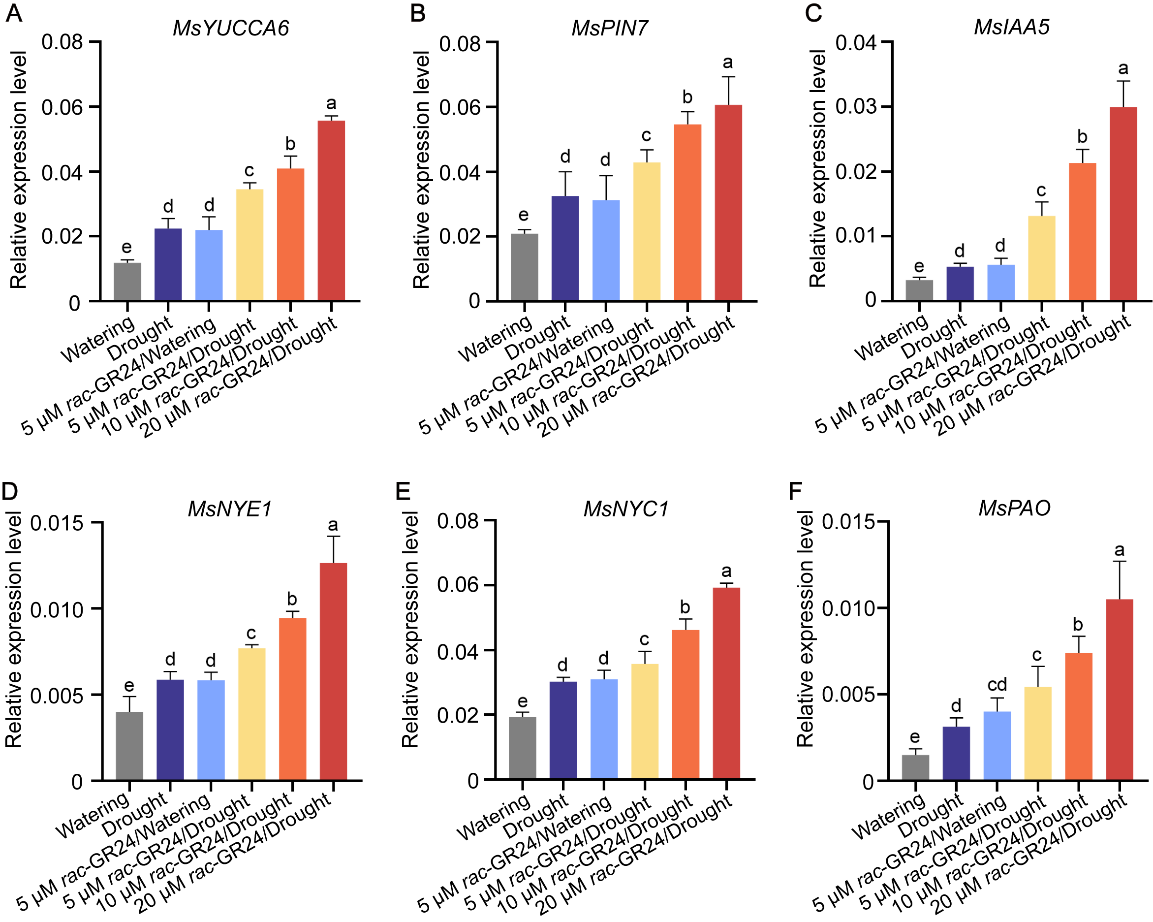


**Figure S14. Effect of SLs on expression of auxin metabolism and leaf senescence. A−C** Expression of auxin metabolism. **D−F** Expression of leaf senescence. Values represent means ± SD. Different letters (a−e) represent significant differences by LSD range test (*P* < 0.05).


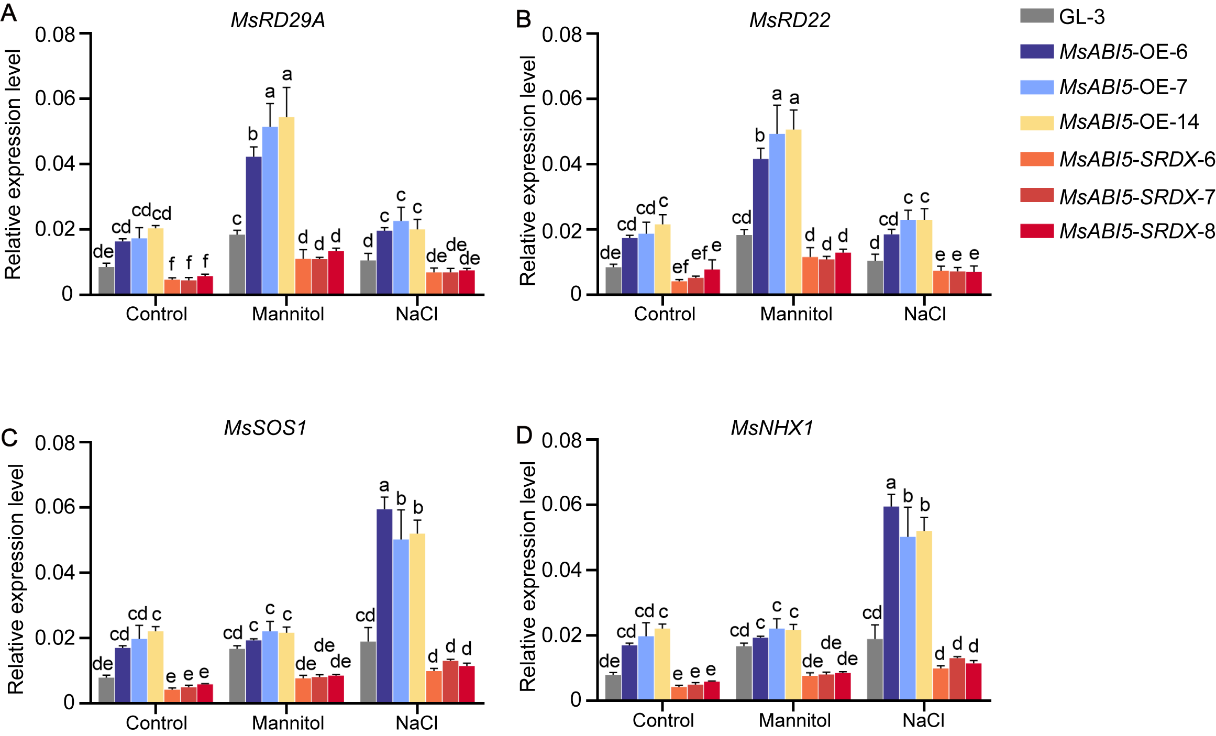


**Figure S15. Effect of MsABI5 on expression of drought and salt stress response genes under mannitol or NaCl condition. A and B** Expression of drought response genes. **C and D** Expression of salt stress response genes. Values represent means ± SD. Different letters (a−f) represent significant differences by LSD range test (*P* < 0.05).


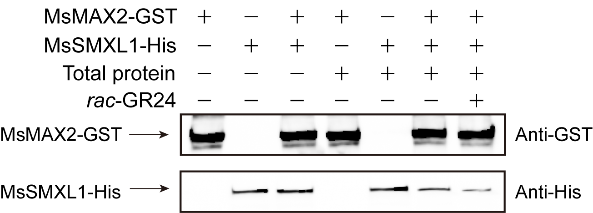


**Figure S16.** MAX2-dependent degradation of MsSMXL1 protein under SLs treatment


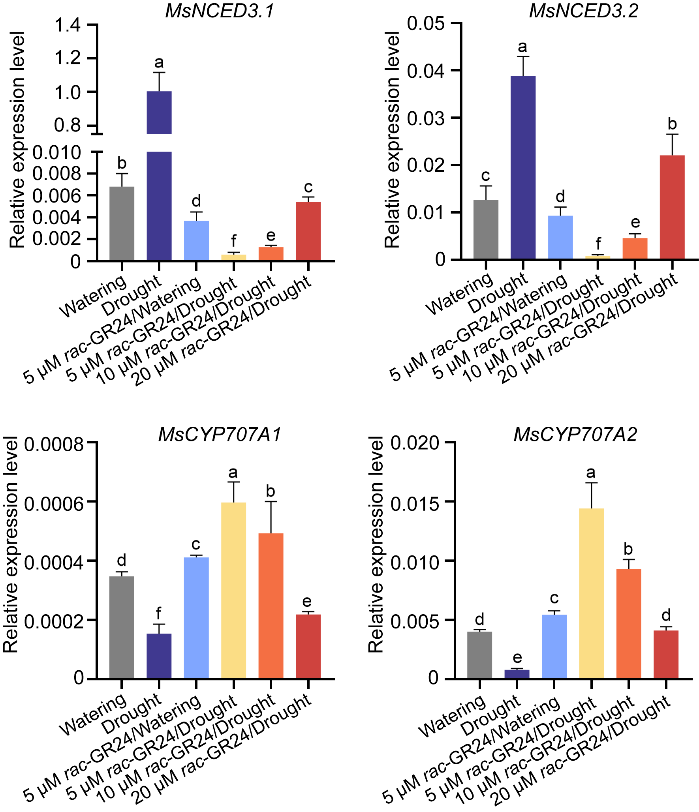


**Figure S17. Effect of *rac*-GR24 on the expression of ABA biosynthesis-related genes and ABA catabolism-related genes.** Values represent means ± SD. Different letters (a−f) indicate significant differences by LSD range test (*P* < 0.05).
