# Supplementary material for: Strigolactones enhance apple drought resistance via the MsABI5-MsSMXL1-MsNAC022 cascade
Source: Hortic Res. 2025 Apr 9;12(7):uhaf101. doi: 10.1093/hr/uhaf101 (PMC12090352; doi:10.1093/hr/uhaf101)
Supplement: Web_Material_uhaf101 [file web_material_uhaf101.zip › Figure S13.pdf]

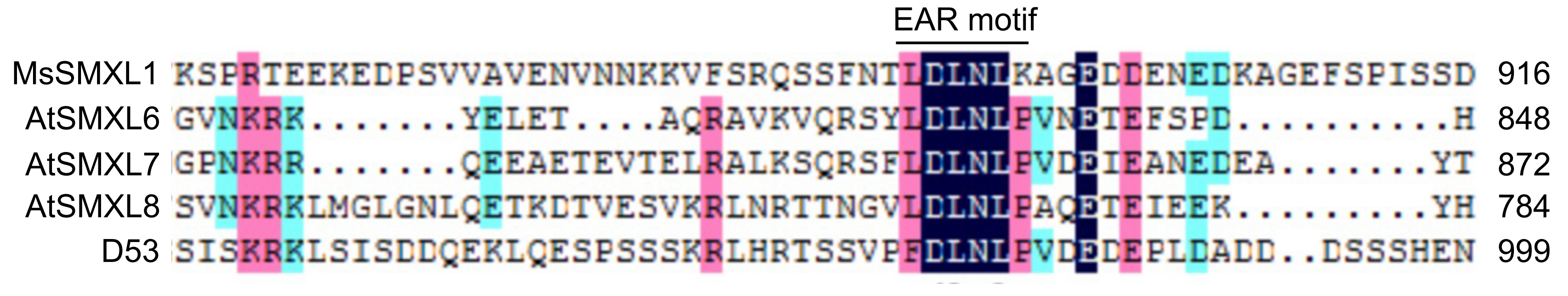
EAR motif
MsSMXL1 KSPRTEEKEDPSVVAVENVNNKKVFSRQSSFNTLDLNLKAGEDDENEDKAGEFSPISSD 916
AtSMXL6 GVNKRK........YELET....AQRAVKVQRSYLDLNLPVNETEFSPD...........H 848
AtSMXL7 GPNKRR........QEEAETEVTELRALKSQRSFLDLNLPVDEIEANEDEA........YT 872
AtSMXL8 SVNKRKLMGLGNLQETKDTVESVKRLNRTTNGVLDLNLPAQETEIEEK.........YH 784
D53 SISKRKLSISDDQEKLQESPSSSKRLHRTSSVPFDLNLPVDEDEPLDADD..DSSSHEN 999
