# Supplementary material for: Strigolactones enhance apple drought resistance via the MsABI5-MsSMXL1-MsNAC022 cascade
Source: Hortic Res. 2025 Apr 9;12(7):uhaf101. doi: 10.1093/hr/uhaf101 (PMC12090352; doi:10.1093/hr/uhaf101)
Supplement: Web_Material_uhaf101 [file web_material_uhaf101.zip › Figure S2.pdf]

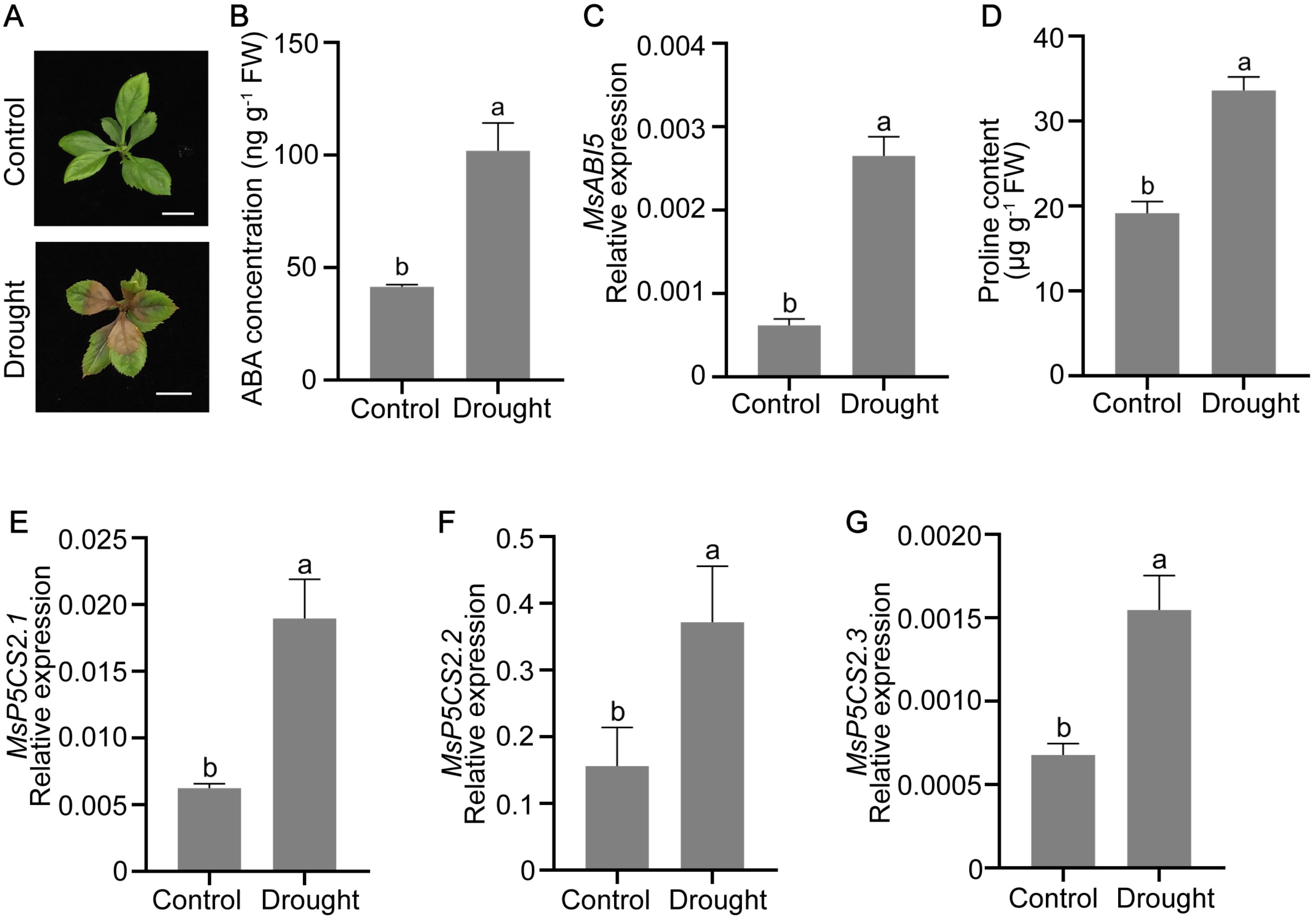

A
Control
Drought
B
ABA concentration (ng g-1 FW)
150
100
50
0
a
b
Control
Drought
C
MsABI5
Relative expression
0.004
0.003
0.002
0.001
0
a
b
Control
Drought
D
Proline content
(μg g-1 FW)
40
30
20
10
0
a
b
Control
Drought
E
MsP5CS2.1
Relative expression
0.025
0.020
0.015
0.010
0.005
0
a
b
Control
Drought
F
MsP5CS2.2
Relative expression
0.5
0.4
0.3
0.2
0.1
0
a
b
Control
Drought
G
MsP5CS2.3
Relative expression
0.0020
0.0015
0.0010
0.0005
0
a
b
Control
Drought
