# Supplementary material for: Strigolactones enhance apple drought resistance via the MsABI5-MsSMXL1-MsNAC022 cascade
Source: Hortic Res. 2025 Apr 9;12(7):uhaf101. doi: 10.1093/hr/uhaf101 (PMC12090352; doi:10.1093/hr/uhaf101)
Supplement: Web_Material_uhaf101 [file web_material_uhaf101.zip › Figure S7.pdf]

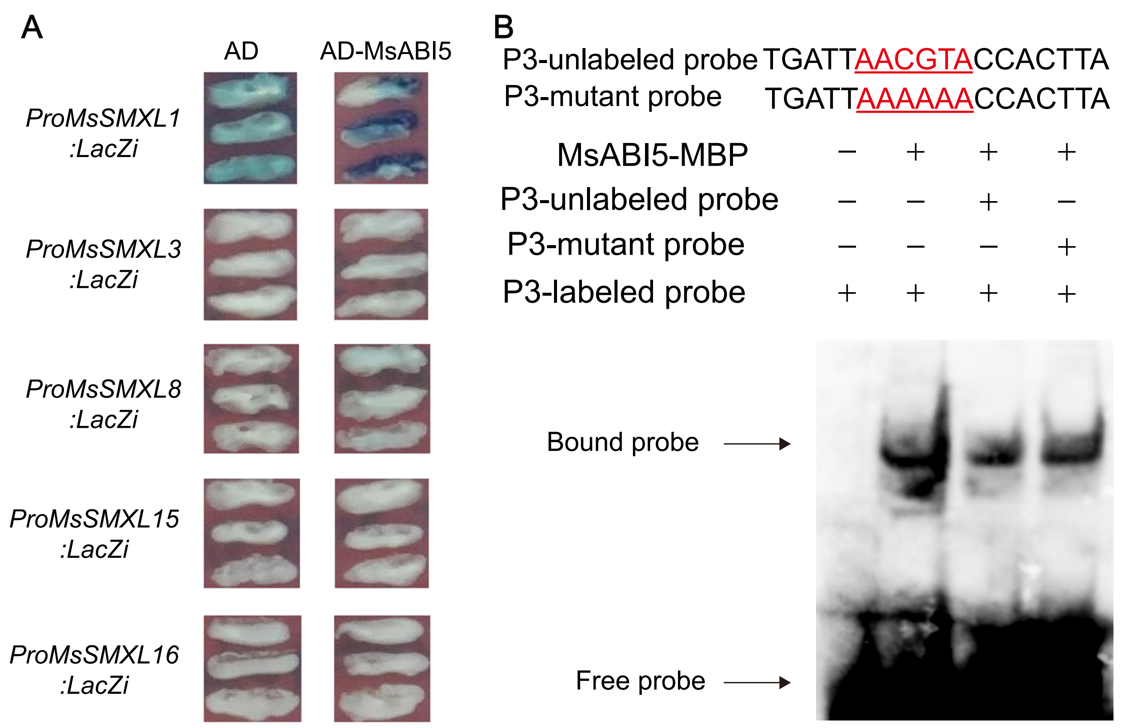

A
AD
AD-MsABI5
ProMsSMXL1 :LacZi
ProMsSMXL3 :LacZi
ProMsSMXL8 :LacZi
ProMsSMXL15 :LacZi
ProMsSMXL16 :LacZi
B
P3-unlabeled probe TGATTAACGTACCACTTA
P3-mutant probe TGATTAAAAAACCACTTA
MsABI5-MBP − + + +
P3-unlabeled probe − − + −
P3-mutant probe − − − +
P3-labeled probe + + + +
Bound probe
Free probe
