# Supplementary figures and images for: Strigolactones enhance apple drought resistance via the MsABI5-MsSMXL1-MsNAC022 cascade
Source: Hortic Res. 2025 Apr 9;12(7):uhaf101. doi: 10.1093/hr/uhaf101 (PMC12090352; doi:10.1093/hr/uhaf101)

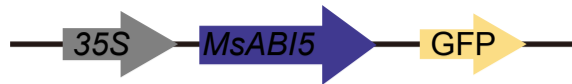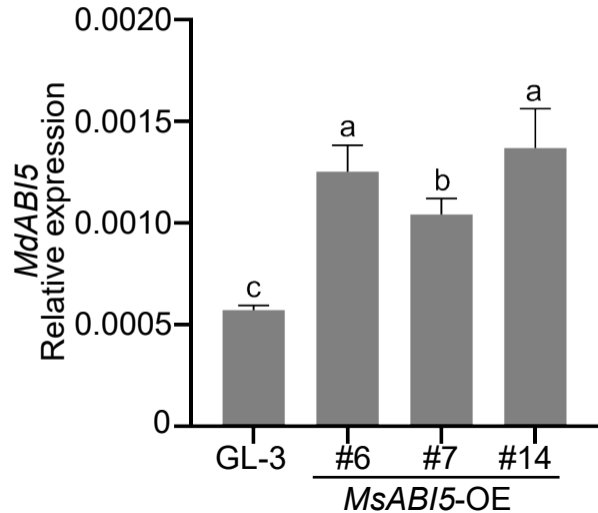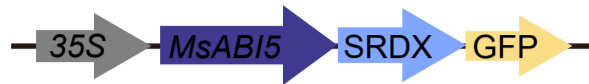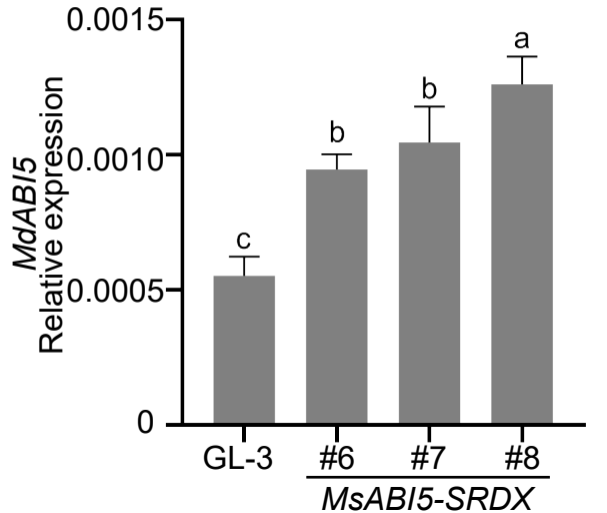

Supplement: Web_Material_uhaf101 [file web_material_uhaf101.zip › Figure S1.pdf]

AD-MsABI5

LexA

LexA-MsSMXL1

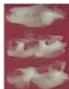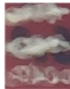

AD

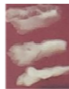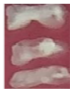

Supplement: Web_Material_uhaf101 [file web_material_uhaf101.zip › Figure S10.pdf]

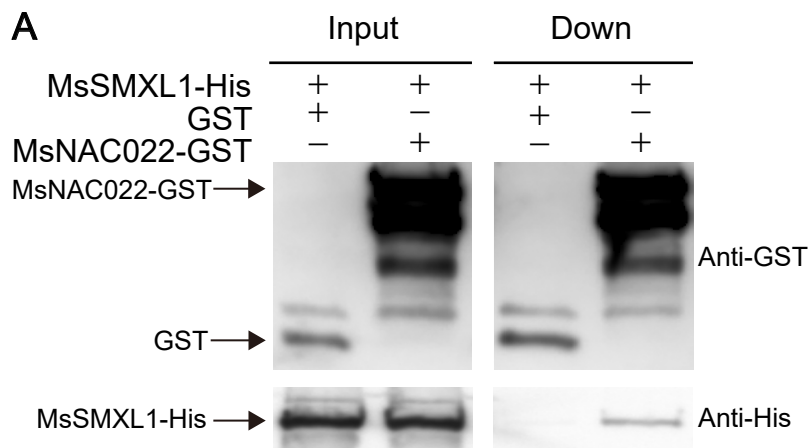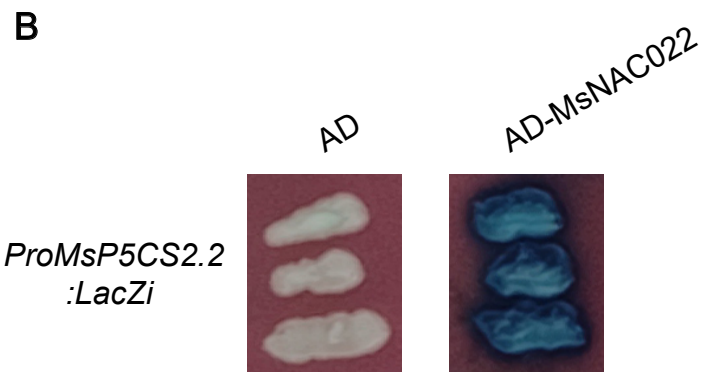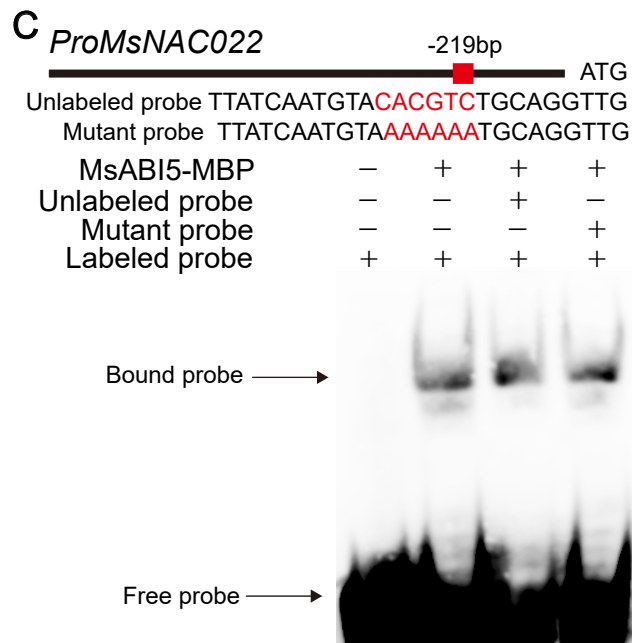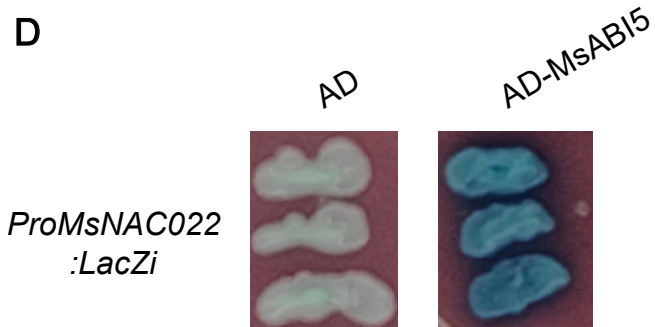

Supplement: Web_Material_uhaf101 [file web_material_uhaf101.zip › Figure S11.pdf]

**A**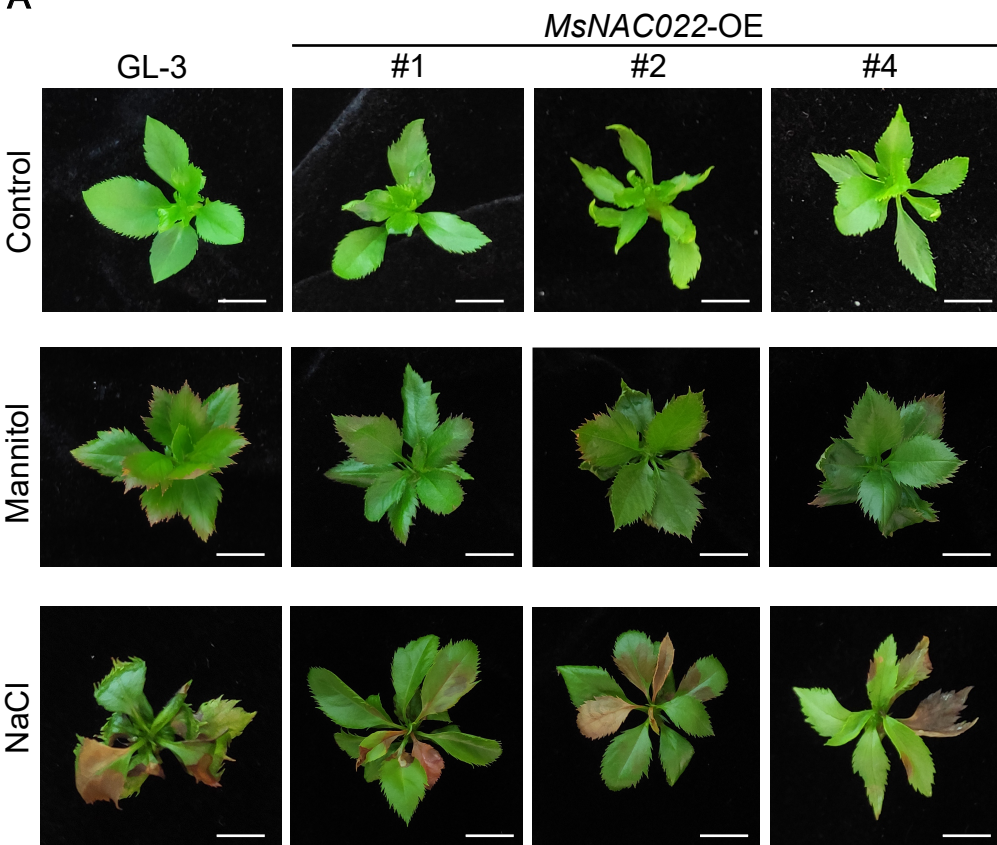**B**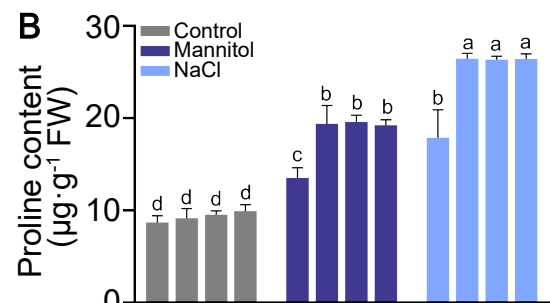**C**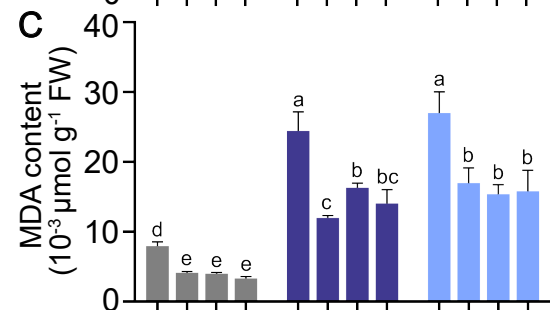**D**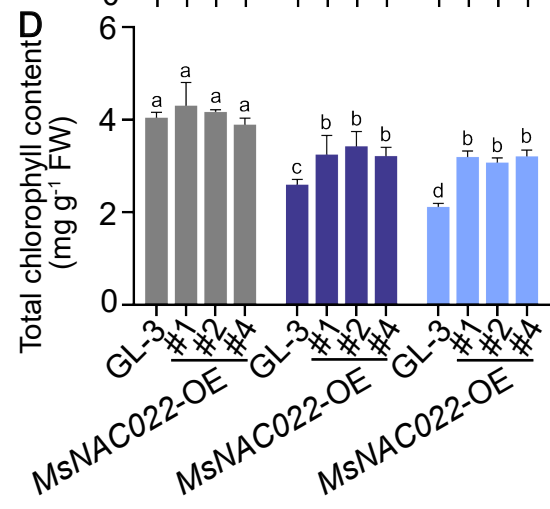

Supplement: Web_Material_uhaf101 [file web_material_uhaf101.zip › Figure S12.pdf]

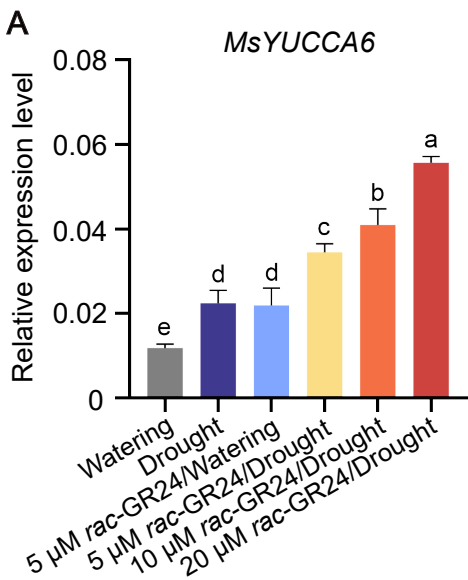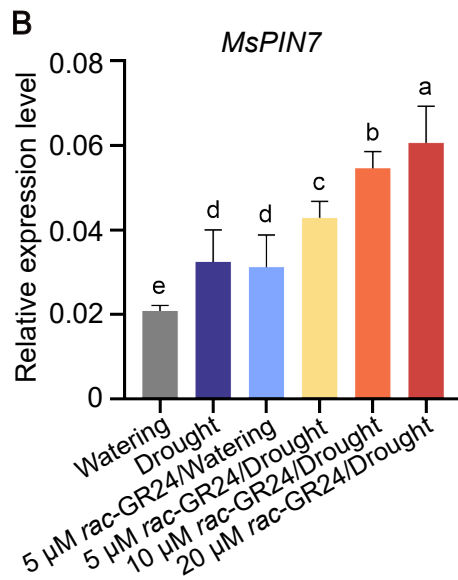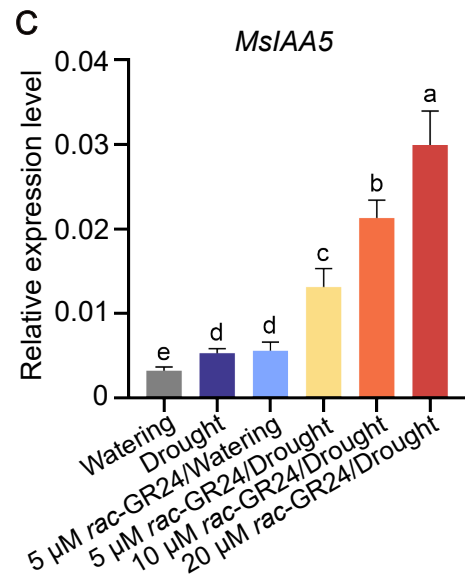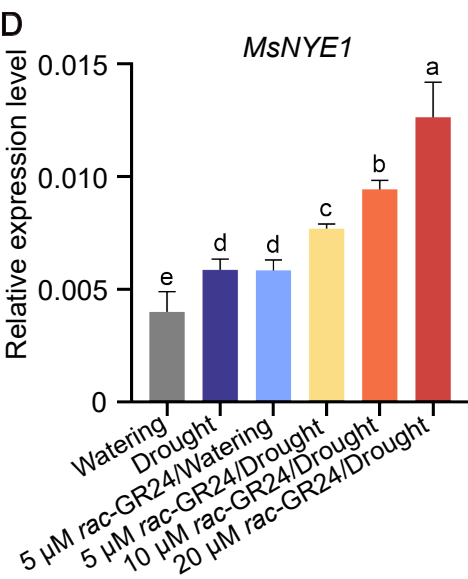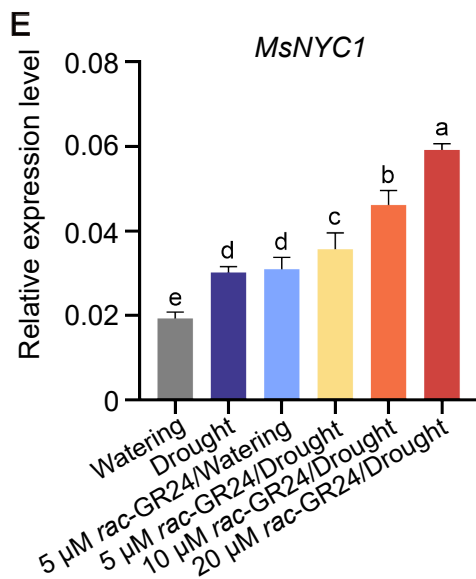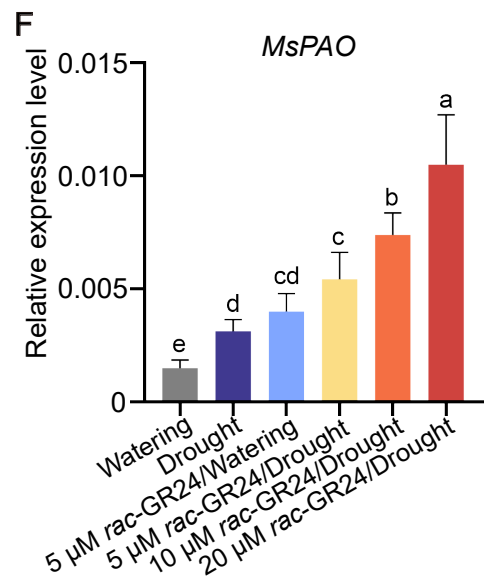

Supplement: Web_Material_uhaf101 [file web_material_uhaf101.zip › Figure S14.pdf]

**A**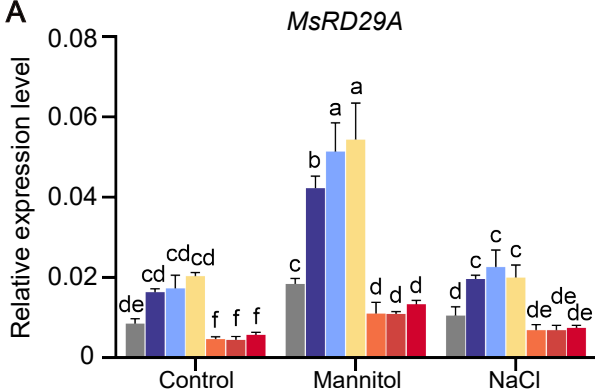**B**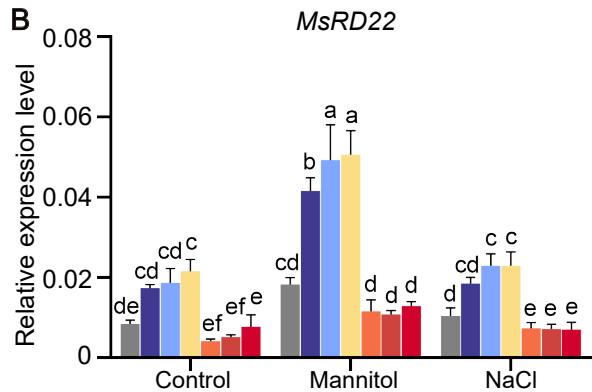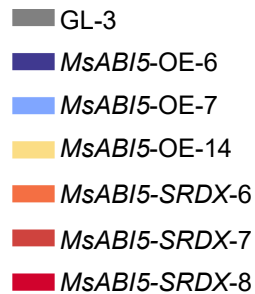**C**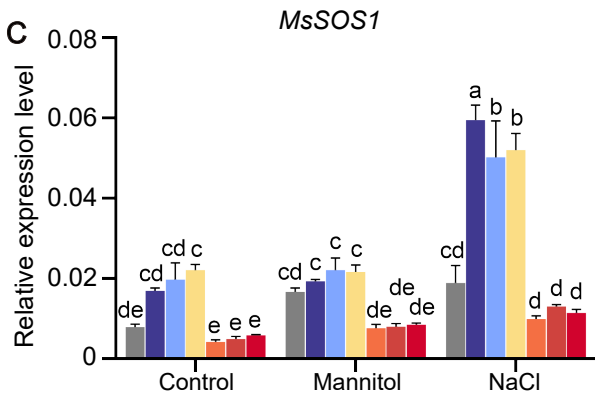**D**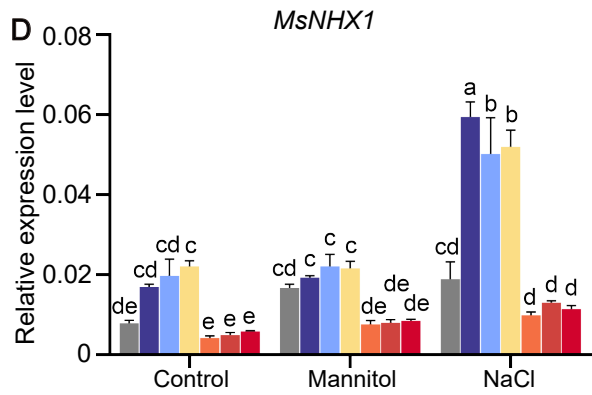

Supplement: Web_Material_uhaf101 [file web_material_uhaf101.zip › Figure S15.pdf]

|                  |   |   |   |   |   |   |   |
|------------------|---|---|---|---|---|---|---|
| MsMAX2.2-GST     | + | — | + | + | — | + | + |
| MsSMXL1-His      | — | + | + | — | + | + | + |
| Total protein    | — | — | — | + | + | + | + |
| <i>rac</i> -GR24 | — | — | — | — | — | — | + |

MsMAX2.2-GST →

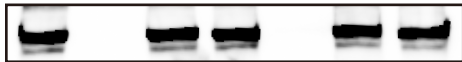

Anti-GST

MsSMXL1-His →

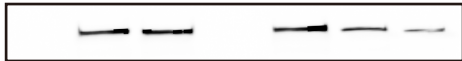

Anti-His

Supplement: Web_Material_uhaf101 [file web_material_uhaf101.zip › Figure S16.pdf]

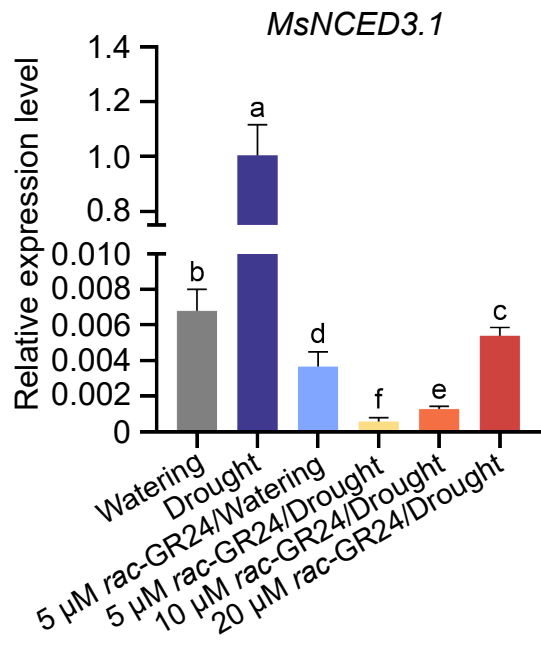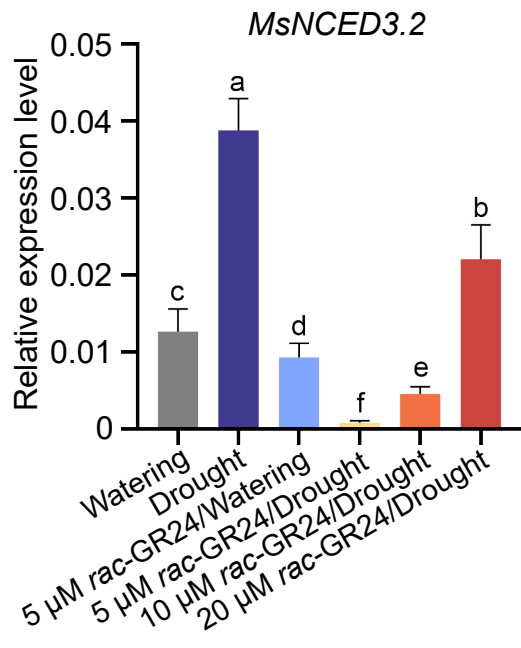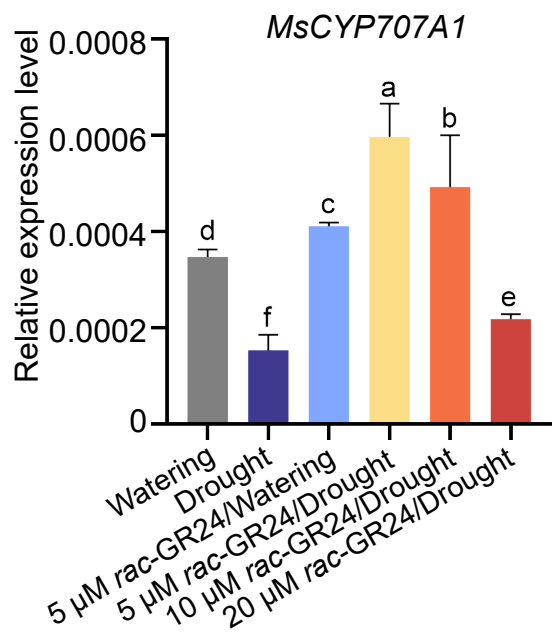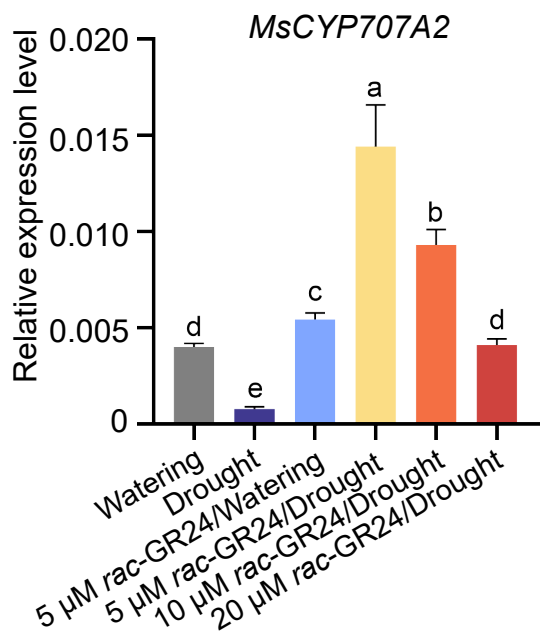

Supplement: Web_Material_uhaf101 [file web_material_uhaf101.zip › Figure S17.pdf]

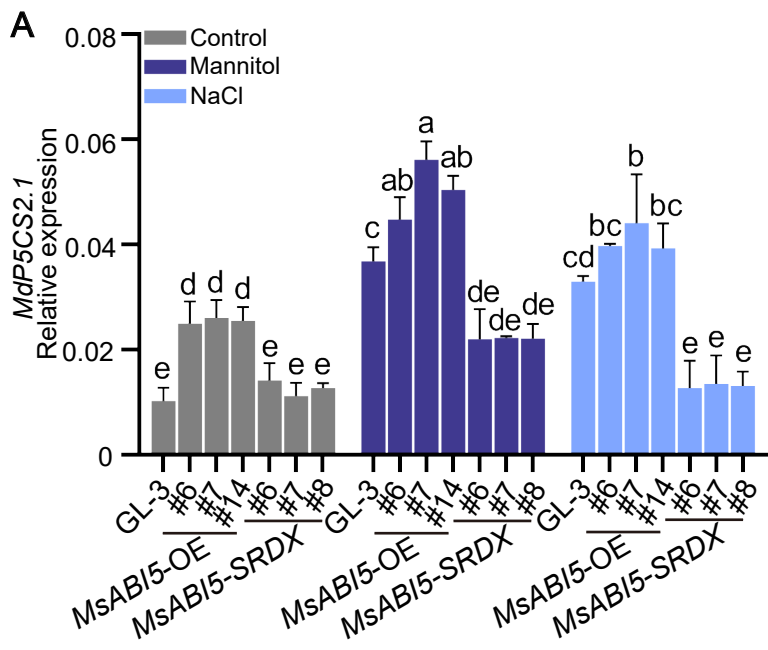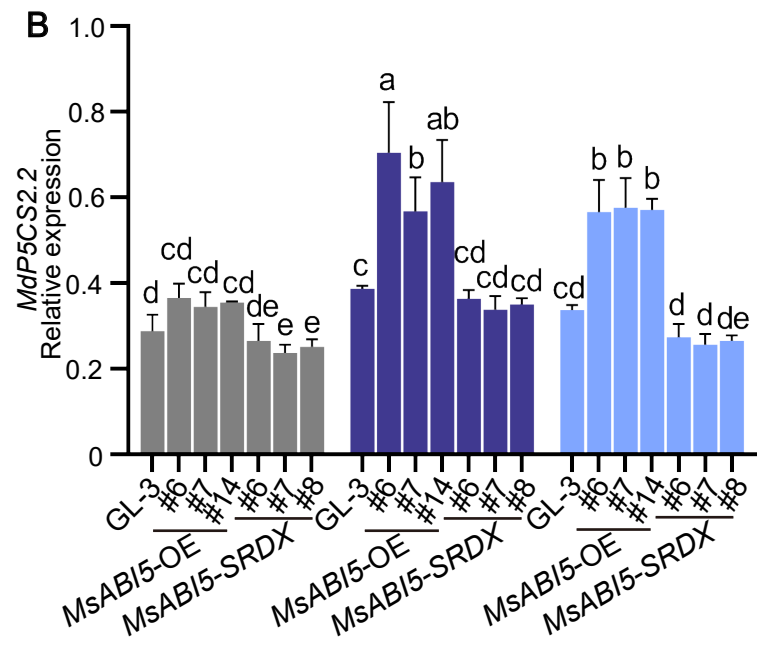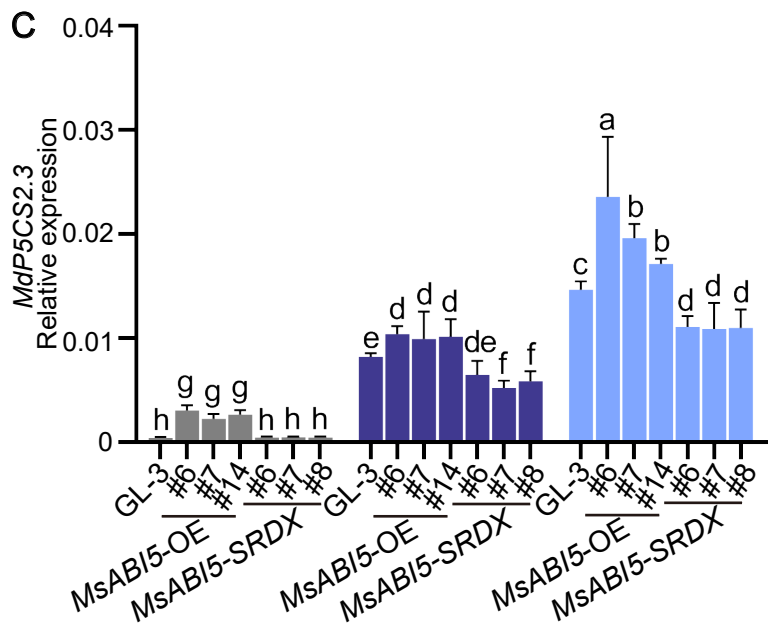

Supplement: Web_Material_uhaf101 [file web_material_uhaf101.zip › Figure S3.pdf]

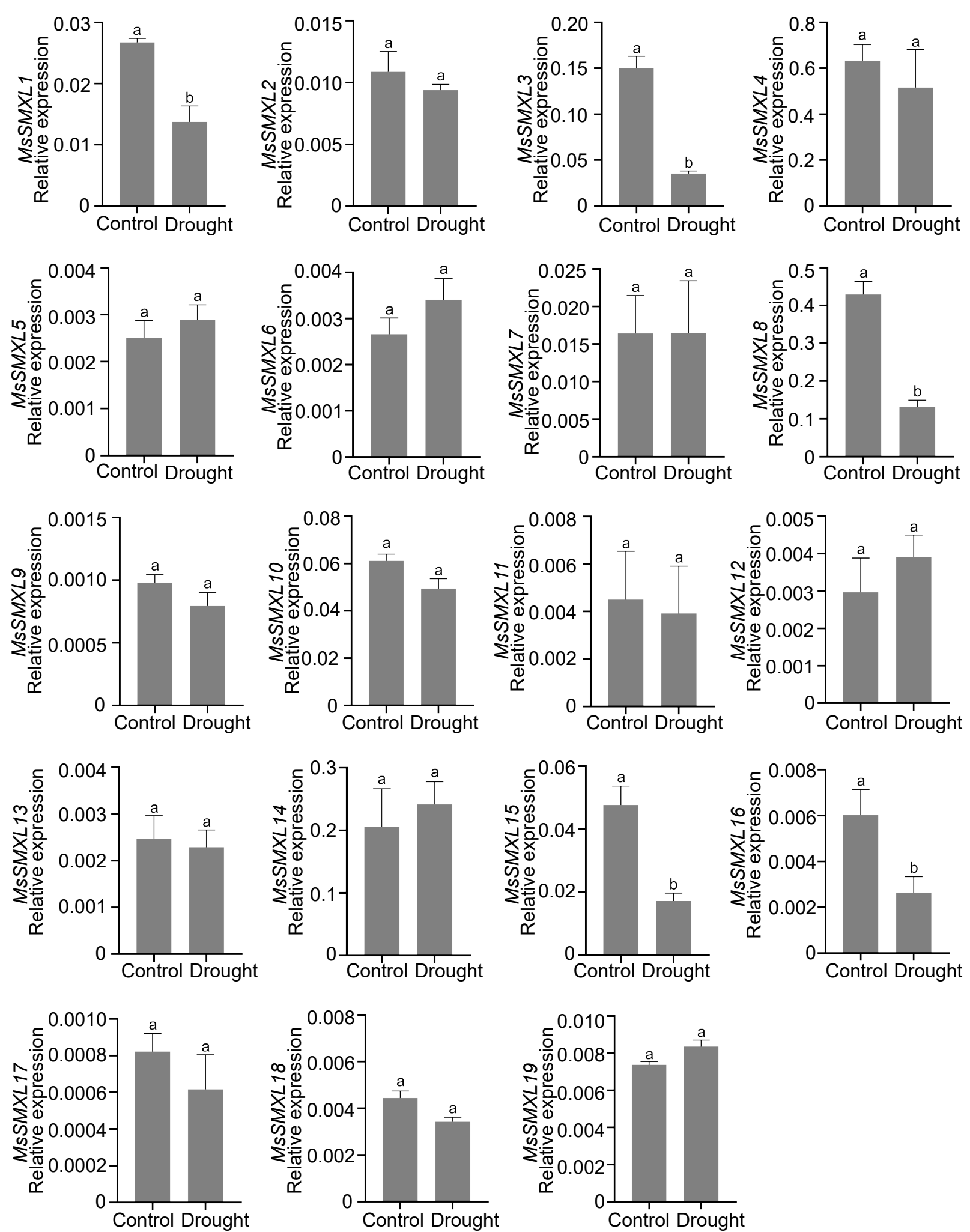

Supplement: Web_Material_uhaf101 [file web_material_uhaf101.zip › Figure S5.pdf]

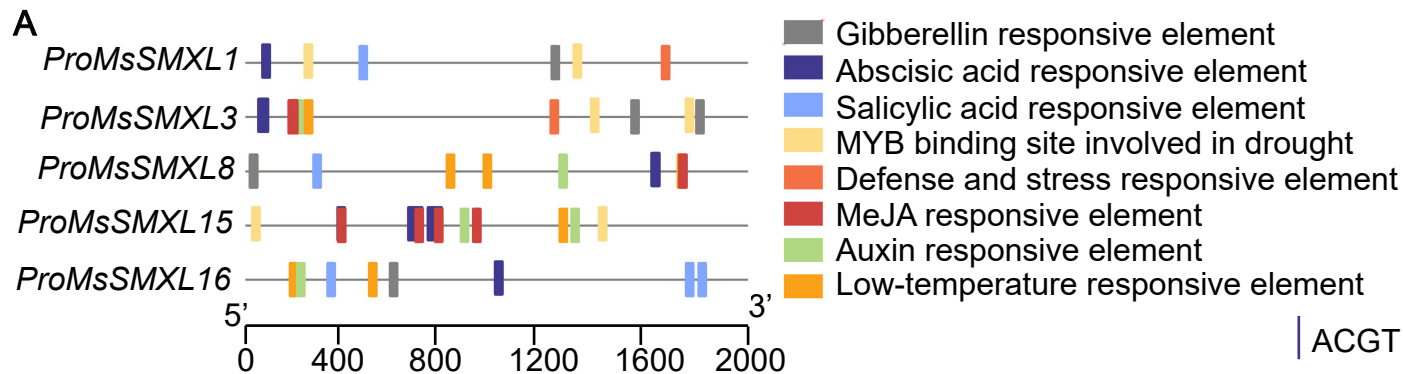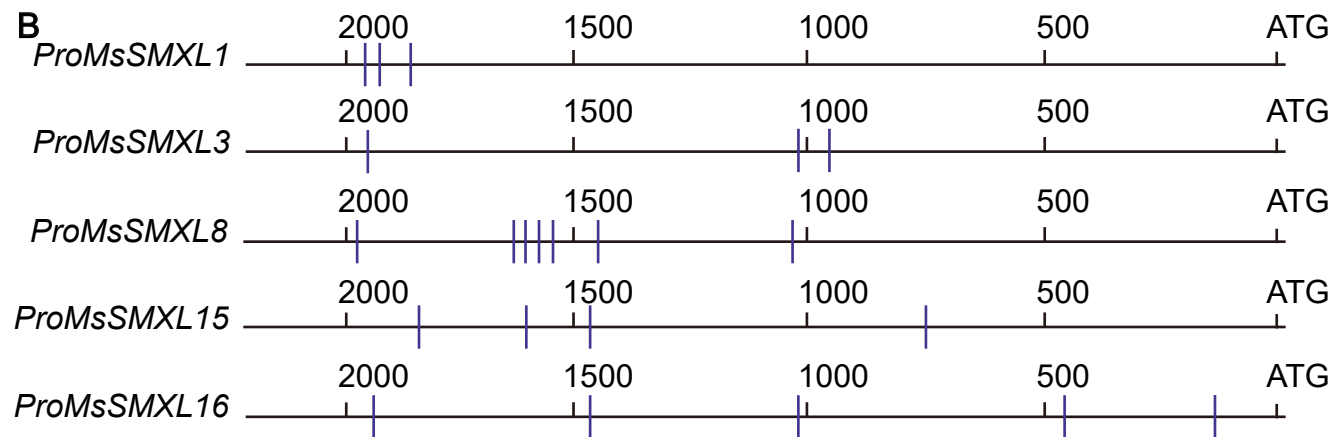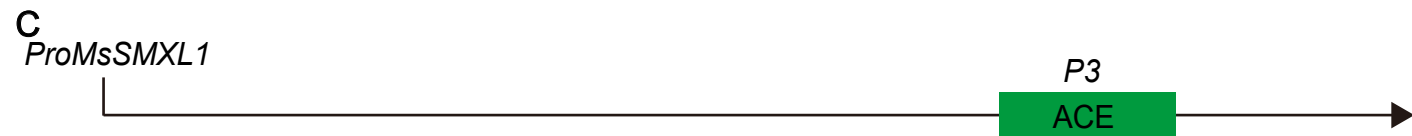

Supplement: Web_Material_uhaf101 [file web_material_uhaf101.zip › Figure S6.pdf]

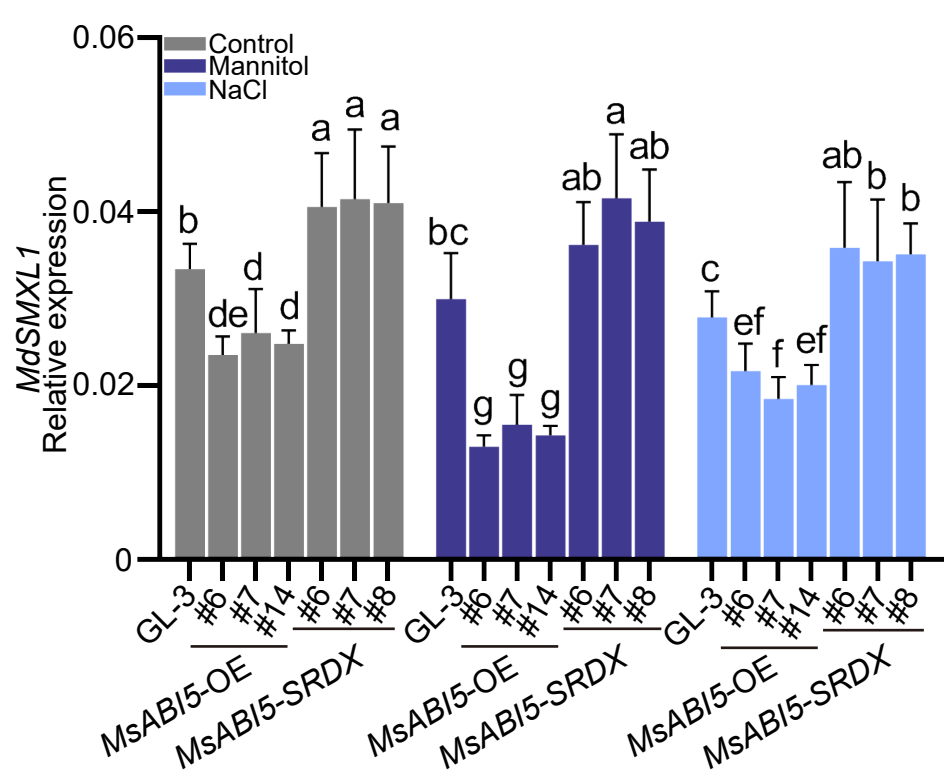

Supplement: Web_Material_uhaf101 [file web_material_uhaf101.zip › Figure S8.pdf]

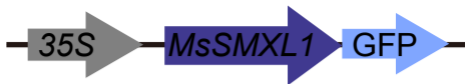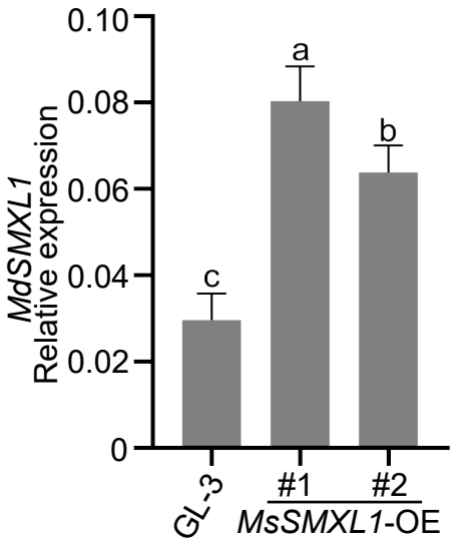

Supplement: Web_Material_uhaf101 [file web_material_uhaf101.zip › Figure S9.pdf]
