# Supplementary material for: Strigolactones enhance apple drought resistance via the MsABI5-MsSMXL1-MsNAC022 cascade
Source: Hortic Res. 2025 Apr 9;12(7):uhaf101. doi: 10.1093/hr/uhaf101 (PMC12090352; doi:10.1093/hr/uhaf101)
Supplement: Web_Material_uhaf101 [file Figure S4.pdf › Figure S4.pdf]

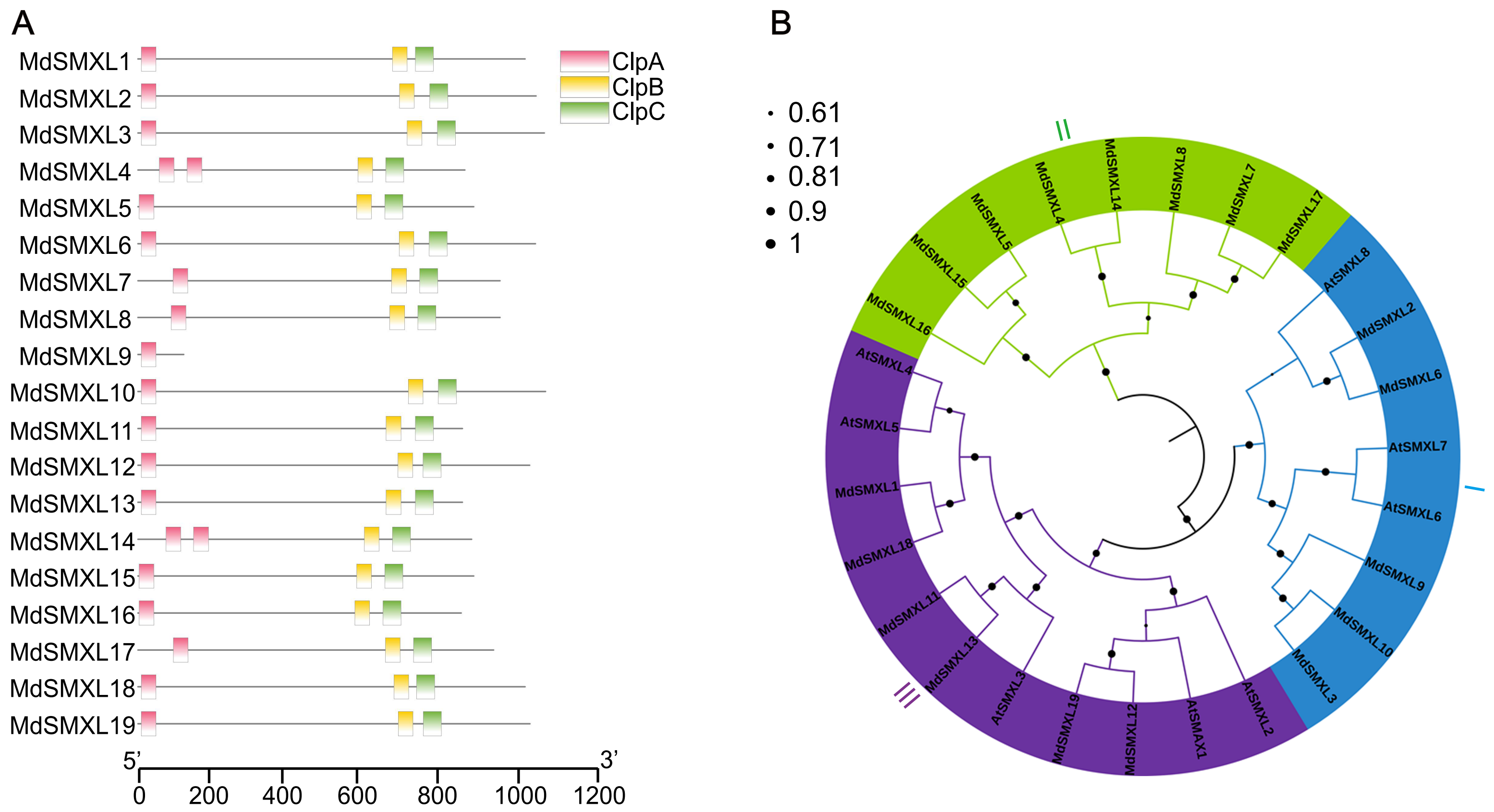
A
MdSMXL1
MdSMXL2
MdSMXL3
MdSMXL4
MdSMXL5
MdSMXL6
MdSMXL7
MdSMXL8
MdSMXL9
MdSMXL10
MdSMXL11
MdSMXL12
MdSMXL13
MdSMXL14
MdSMXL15
MdSMXL16
MdSMXL17
MdSMXL18
MdSMXL19
ClpA
ClpB
ClpC
5'
3'
0
200
400
600
800
1000
1200
B
0.61
0.71
0.81
0.9
1
I
II
III
MdSMXL16
MdSMXL15
MdSMXL5
MdSMXL4
MdSMXL14
MdSMXL8
MdSMXL7
MdSMXL17
AtSMXL8
MdSMXL2
MdSMXL6
AtSMXL7
AtSMXL6
MdSMXL9
MdSMXL10
MdSMXL3
AtSMXL2
AtSMAX1
MdSMXL12
MdSMXL19
AtSMXL3
MdSMXL13
MdSMXL11
MdSMXL18
MdSMXL1
AtSMXL5
AtSMXL4
